# Transcriptomic analysis reveals the key immune-related signalling pathways of *Sebastiscus marmoratus* in response to infection with the parasitic ciliate *Cryptocaryon irritans*

**DOI:** 10.1186/s13071-017-2508-7

**Published:** 2017-11-21

**Authors:** Fei Yin, Dong Qian

**Affiliations:** 1 Key Laboratory of Applied Marine Biotechnology, Ministry of Education, Ningbo University, Ningbo, 315211 China; 2 Collaborative Innovation Center for Zhejiang Marine High-efficiency and Healthy Aquaculture, Ningbo University, Ningbo, 315211 China

**Keywords:** *Sebastiscus marmoratus*, *Cryptocaryon irritans*, Transcriptome, Immune system, Signalling pathway

## Abstract

**Background:**

False kelpfish (*Sebastiscus marmoratus*) is one of the target species in artificial breeding in China, and is susceptible to infection by *Cryptocaryon irritans*, which is an obligate parasitic ciliate that lives in the epithelium of the fish gills, skin and fins. Here, we sought to understand the mechanisms of molecular immunity of *S. marmoratus* against *C. irritans* infection.

**Methods:**

We carried out an extensive analysis of the transcriptome of *S. marmoratus* immune-related tissues. A paired-end library was constructed from the cDNA synthesized using a Genomic Sample Prep Kit. Five normalized cDNA libraries were constructed using RNA from the control group and the four groups of *C. irritans*-infected fish. The libraries were sequenced on an Illumina Mi-Seq platform, and functional annotation of the transcriptome was performed using bioinformatics software.

**Results:**

The data produced a total of 149,983,397 clean reads from five cDNA libraries constructed from *S. marmoratus* immune-related tissues. A total of 33,291 unigenes were assembled with an average length of 1768 bp. In eggNOG (Evolutionary Genealogy of Genes: non-supervised orthologous groups) categories, 333 unigenes (0.94%) were assigned to defense mechanisms. In the immune system process sub-categories of gene ontology (GO) enrichment analysis, with the passage of time post-infection, the number of differentially expressed genes (DEGs) was reduced from 24 h to 48 h but then increased from 72 h to 96 h. Specifically, the immune-related differentially expressed genes (IRDEGs), which belong to the KEGG (Kyoto encyclopedia of genes and genomes) pathways, such as the complement and coagulation cascades, chemokine signalling pathways and toll-like receptor signalling pathways were mainly observed at 24 h post-infection.

**Conclusions:**

Infection with *C. irritans* resulted in a large number of DEGs in the immune-related tissues of *S. marmoratus*. The rapid and significant response of the *S. marmoratus* immune signalling pathways following *C. irritans* infection may be associated with their involvement in the immune process.

**Electronic supplementary material:**

The online version of this article (10.1186/s13071-017-2508-7) contains supplementary material, which is available to authorized users.

## Background

As a common target species for the fishing industry, the false kelpfish (*Sebastiscus marmoratus*) is mainly distributed in the Western North Pacific. The small size and ease of feeding have made *S. marmoratus* a target species in artificial breeding programs in China. The widespread occurrence of the marine parasitic disease cryptocaryoniasis has resulted in significant limitations in the aquaculture of these fishes. For example, available data indicate that *S. marmoratus* is a susceptible host to *Cryptocaryon irritans* [1]. For these reasons, this species of fish is a good candidate in which to study the aetiology and pathogenesis of *C. irritans*. For instance, Sun et al. (2011) first successfully constructed a subculture system of *C. irritans* with *S. marmoratus* as the host [2]. Previous research has indicated that *C. irritans* infection results in elevated of serum cortisol, glucose contents, accelerate respiratory rate, and reduced food intake in *S. marmoratus* [1, 3]. However, to date, the patterns of immunological responses in infected *S. marmoratus* has not been thoroughly studied.

*Cryptocaryon irritans* is an obligate parasitic ciliate that lives in the epithelium of the fish gills, skin and fins, infecting most marine teleosts. To investigate the mechanisms of immune response to *C. irritans*, extensive studies from the physiological and biochemical perspectives at the molecular level have been conducted [4–6]. Recent work has indicated that the infection with sub-lethal doses of *C. irritans*, or the injection of inactivated *C. irritans* cells, could not only significantly improve the fish’s mucus production and serum antibody titers but also increase the activities of mucus LZM and AKP. Also, the contents of mucus IgM and complement C3, and the protective immune rate are different [7–9]. In recent years, numerous data have been produced regarding gene-level regulation that suggests that immune-related (IR) factors such as *TLR2*, *MyD88*, *IL1β* [10], *Pc-pis* [11], *TNF-α*, *MHC I/II*, and *TGFβ1* [12], *NCCRP1* [13], *TRAF6* [14], *IRAK4* [15], *IL34/MCSF2*, *MCSFR1/MCSFR2* [16], *Nrdp1* [17] and *Interferon-γ* [18] in the fish are linked to *C. irritans* infection. Despite this work, the changes in the relative amounts of a few factors are not enough to provide a thorough understanding of the immune responses in infected fish.

A transcriptome is the set of all RNA transcripts in the cells. Transcriptome analysis indicates the level of mRNA of a specific gene in response to stimuli. From this evaluation of the expression (an up or down level of each RNA), functions and activated pathways may be inferred. In recent years, transcriptome analysis has been applied in the studies on the interactions between fish and parasites [19, 20]. To have a better understanding of *C. irritans* infection and immunological response of the host, Khoo et al. (2012) developed a cDNA microarray analysis to analyze the stress response of Asian sea bass (*Lates calcarifer*) to *C. irritans* infection [21]. With the development of next-generation high-throughput sequencing technique, RNA-seq has also been applied in the studies of the fish-*C. irritans* interaction. Two recent reports have examined the transcriptome of *C. irritans* infected large yellow croaker (*Larimichthys crocea*) in IR tissues through RNA-seq [6, 22], and a transcriptomic variation analysis on the *C. irritans* locally-infected skin of orange-spotted grouper (*Epinephelus coioides*) has been reported [23]. These data provide a frame of reference to enhance our understanding of the mechanisms of immunity of fish to *C. irritans* infection at the molecular level, by combining a variety of bioinformatic analyses, such as evolutionary genealogy of genes: non-supervised orthologous groups (eggNOG), gene ontology (GO), and Kyoto encyclopedia of genes and genomes (KEGG). However, there have been no published studies examining the responses using this approach in other fish species. Furthermore, little is known about the variations in the differentially expressed genes (DEGs) enriched signalling pathways over time following *C. irritans* infection.

In response to *C. irritans* infection, skin, gills, head kidney, spleen and liver are believed to be the most important immune responsive tissues [17]. Skin showed a local immune response against *C. irritans* infection [23]. Liver from *C. irritans*-immunized *L. crocea* was the first tissue examined with respect to a comparative gene transcription analysis [22]. In the present study, *S. marmoratus* were infected with *C. irritans* theronts at a sub-lethal concentration [1]. RNA-seq was utilized to detect the transcripts in three pooled systemic immune-related tissues of *S. marmoratus* at 24, 48, 72 and 96 h post-infection utilizing bioinformatics analysis of the IR-pathways, as well as comparing the variations of all the involved DEGs. Key genes and signalling pathways that are involved as potential regulatory role were further explored.

## Methods

### Parasite and experimental fish

*Cryptocarion irritans* were obtained from naturally infected Pompano (*Trachinotus ovatus*, 500 ± 50 g), and *T. ovatus* were then used as the model to establish the passage system. Propagation and collection of tomonts and theronts were conducted as previously described [24].

*Sebastiscus marmoratus* (45 ± 3 g) were purchased from local fisheries in Aaotou, Huizhou City, Guangdong Province, China. No *C. irritans* trophonts were detected on the gills, skin, or fins of these fish, and no immobilization occurred when *C. irritans* theronts were incubated in fish blood serum. The fish were overfed twice daily (8:00 and 15:00) with mixed wild fish meat that was purchased and stored at -20 °C until feeding [1]. The water temperature, salinity, light intensity, and photoperiod for aquaculture were 26 ± 1 °C, 29–31%, 1000 Lux, and 12 L:12D, respectively.

### Experimental design and sample collection

Active *C. irritans* theronts released from tomonts within 2 h were collected, and the concentration of theronts was calculated [25]. Sixty healthy fish were challenged with theronts at a dose of 5000 theronts per fish (96 h after the infection at 5000 theronts/fish, the survival of *S. marmoratus* was 85%), as previously described [1]. Spleens, head kidneys and livers of 3 fish were sampled for expression analysis at 24 h (group B), 48 h (group C), 72 h (group D), and 96 h (group E) each time point post-challenge and unchallenged fish were sampled as control (group A). The tissues were immediately placed in Sample Protector for RNA/DNA (TaKaRa, Dalian, China) and then stored at -20 °C until RNA extraction.

### Extraction of total RNA and sample preparation for RNA-Seq

Total RNA was extracted using Trizol Reagent® (Invitrogen, Carlsbad, CA, USA) following the manufacturer’s instructions, and then treated with RNase-free DNase I included with the kit. The RNA concentration of each sample was quantified using an Agilent 2100 Bioanalyzer (Agilent technologies, Santa Clara, USA). Quantity, purity and integrity were determined with a 1.2% (*w*/*v*) agarose gel and with a Nanodrop-1000 spectrophotometer (NanoDrop, Wilmington, DE, USA). High-quality RNA with absorbance ratios at 260 nm/280 nm > 1.9 was selected for high-throughput sequencing. The extracted total RNA was resuspended in distilled water and stored at -80 °C before use. To obtain the transcriptome data of fish without tissue specificity, different tissues were pooled in equal amounts of RNA as previously described [6, 26, 27]. In this study, each RNA sample was collected from 3 different IR-tissues, and each tissue sample contained a mixture of samples obtained from 3 fish of the same treatment condition. The purified mRNA was then enriched with oligo (dT) conjugated to magnetic beads and fragmented using divalent cations under elevated temperature.

Random primers and reverse transcriptase were used for first strand cDNA synthesis, and the second strand cDNA was synthesized using DNA polymerase I and RNase H (Invitrogen). A paired-end library was constructed from the cDNA synthesized using the Genomic Sample Prep Kit (Illumina, San Diego, CA, USA). Five normalized cDNA libraries were constructed using RNA from groups A-E. The libraries were sequenced on an Illumina Mi-Seq platform.

### Assembly and functional annotation of the transcriptome

A paired-end (PE) sequencing strategy was used to improve the assembly of the entire transcriptome de novo. Raw PE reads with an average length of 250 bp were generated. The FastQC program (http://www.bioinformatics.babraham.ac.uk/projects/ fastqc/) was used to trim adaptor sequences and remove low-quality sequences (defined as when the percentage of bases with a quality value ≤5 exceeds 50% in the read). A custom Perl program was used to remove short sequences (< 50 bp). Trinity (https://github.com/trinityrnaseq/ trinityrnaseq/wiki) was used to perform the de novo assembles, and the resulting high-quality sequences were assembled into contigs and transcripts [28]. To reduce data redundancy, TGICL was used to assemble and cluster transcripts with a minimum length of 200 bp. The longest sequences in each cluster were reserved and designated as unigenes [29]. GO annotation based on BLASTx hits with the NCBI Nr database was performed using Blast2GO (E-value <10^−5^) (https://www.blast2go.com/). The expression levels of differentially expressed unigenes were annotated using KO analysis (http://www.genome.jp/kegg/tool/map_pathway2.html). KEGG automatic annotation server (KASS) was used with the default parameters to perform pathway annotation as described previously [27].

### Identification of differently expressed genes

The clean reads from each of the five libraries (A, B, C, D and E) were mapped back to the transcriptome assembly using the Bowtie2 software with default settings. The number of reads aligned to each unigene in the alignment file was determined for each sample. The number of each transcript aligned to a gene was then normalized and calculated using uniquely mapped reads by the RPKM (Reads per kilobase of transcripts per million fragments mapped) method [30]. The differential expression analysis identified DEGs among the five different groups listed above was performed using the DESeq web tool (http://www-huber.embl.de/users/anders/ DESeq). The false discovery rate (FDR) method was used to identify the significance of differences in gene expression. Unigenes with fold changes >2 and an adjusted *P*-value <0.05 were considered to be differentially expressed genes [31]. The dispersion factor can be understood as the square of the coefficient of biological variation (Additional file 1: Table S1).

### Quantitative RT-PCR (qRT-PCR) verification

To validate the RNA-Seq (Quantification) results, eight genes were randomly selected for qRT-PCR analysis using a SYBR Premix Ex *Taq* kit (Invitrogen). The RNA-Seq data indicated that 24 h post-infection is the peak time-point for the host response to *C. irritans* infection. Thus, group B and the control group were used for the qRT-PCR verification assay. RNA samples used were the same that were used for Illumina library synthesis. The specific primers used are listed in the Additional file 2: Table S2, and *β-actin* was used as an internal control. All reactions were performed using technical triplicates. The thermal profile for SYBR Green RT-PCR was 95 °C for 5 min, followed by 40 cycles of 95 °C for 15 s, and 60 °C for 30 s. The relative expression levels of target genes were analyzed using the comparative threshold (CT) cycle method as previously described [32]. The RT-qPCR data were analyzed using one-way analysis of variance (ANOVA).

## Results and discussion

### Sequencing and de novo transcriptome assembly

**Fig. 1:**
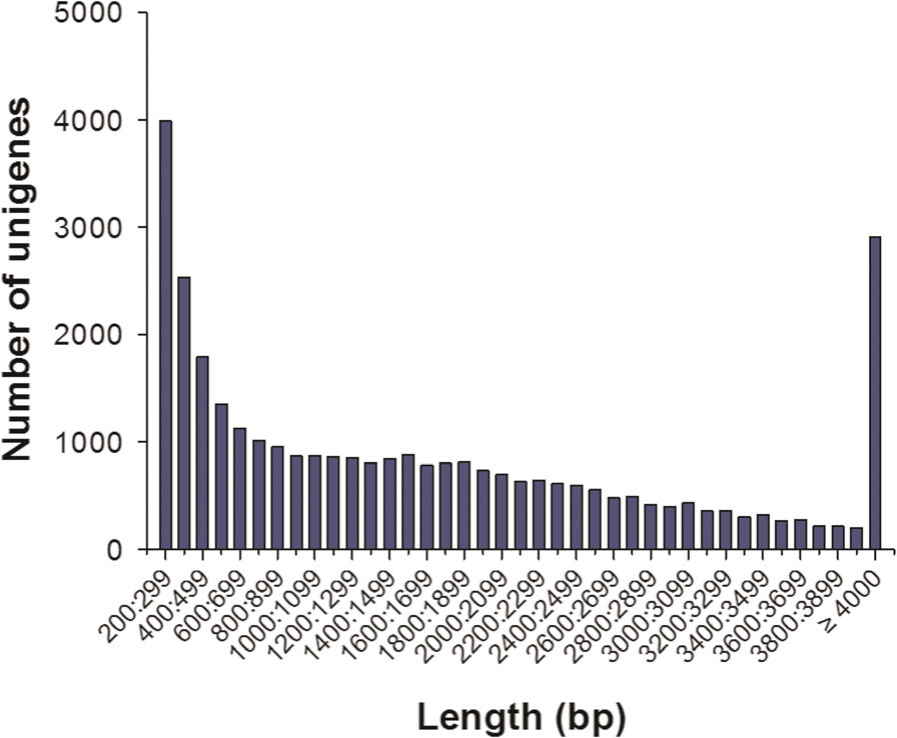
Transcriptome sequence length distributions of *S. marmoratus* unigenes. The x-axis indicates unigene size and the y-axis indicates the number of unigenes with different lengths

The assembled transcriptome consisted of a total of 165,442,302 paired-end raw reads with an average length of 101 bp (Additional file 3: Table S3). A total of 149,983,397 clean reads were obtained for subsequent analysis (Additional file 3: Table S3). Low-quality sequences and adaptor sequences were eliminated from the original data sequence by quality analysis. De novo transcriptome assembly was performed for the clean reads using Trinity. A summary of all contigs, transcripts and unigene assemblies are presented in Table 1. The total length and number of contigs were 147,180,023 bp and 434,694, respectively. The maximum contig length detected was 28,497 bp, with an average length of 338.58 bp (N50:524). The observed GC content was 44.40%. The total length and number of transcripts were 197,518,635 bp and 213,321, respectively. The maximal length of the transcript was 28,455 bp with an average length of 926 bp (N50: 1976) and had a GC content of 46.57%. The total length and number of unigenes were 58,850,763 bp and 33,291, respectively. The maximal unigene length was 28,455 bp with an average length of 1768 bp (N50: 2756), and a GC content of 49.66% (Table 1). The unigene length distribution is displayed in Fig. 1. The majority of sequences ranged from 200 to 299 bp in length. There were 2904 unigenes with length above 4000 bp.

**Table 1:** Summary of de novo assembly of transcriptomic profiles of *Sebastiscus marmoratus*

	Total length (bp)	Sequence No.	Max length (bp)	Average length (bp)	N50	> N50 reads No.	GC (%)
Contig	147,180,023	434,694	28,497	338.58	524	51,710	44.40
Transcript	197,518,635	213,321	28,455	926	1976	27,719	46.57
Unigene	58,850,763	33,291	28,455	1768	2756	6903	49.66

### EggNOG

**Fig. 2:**
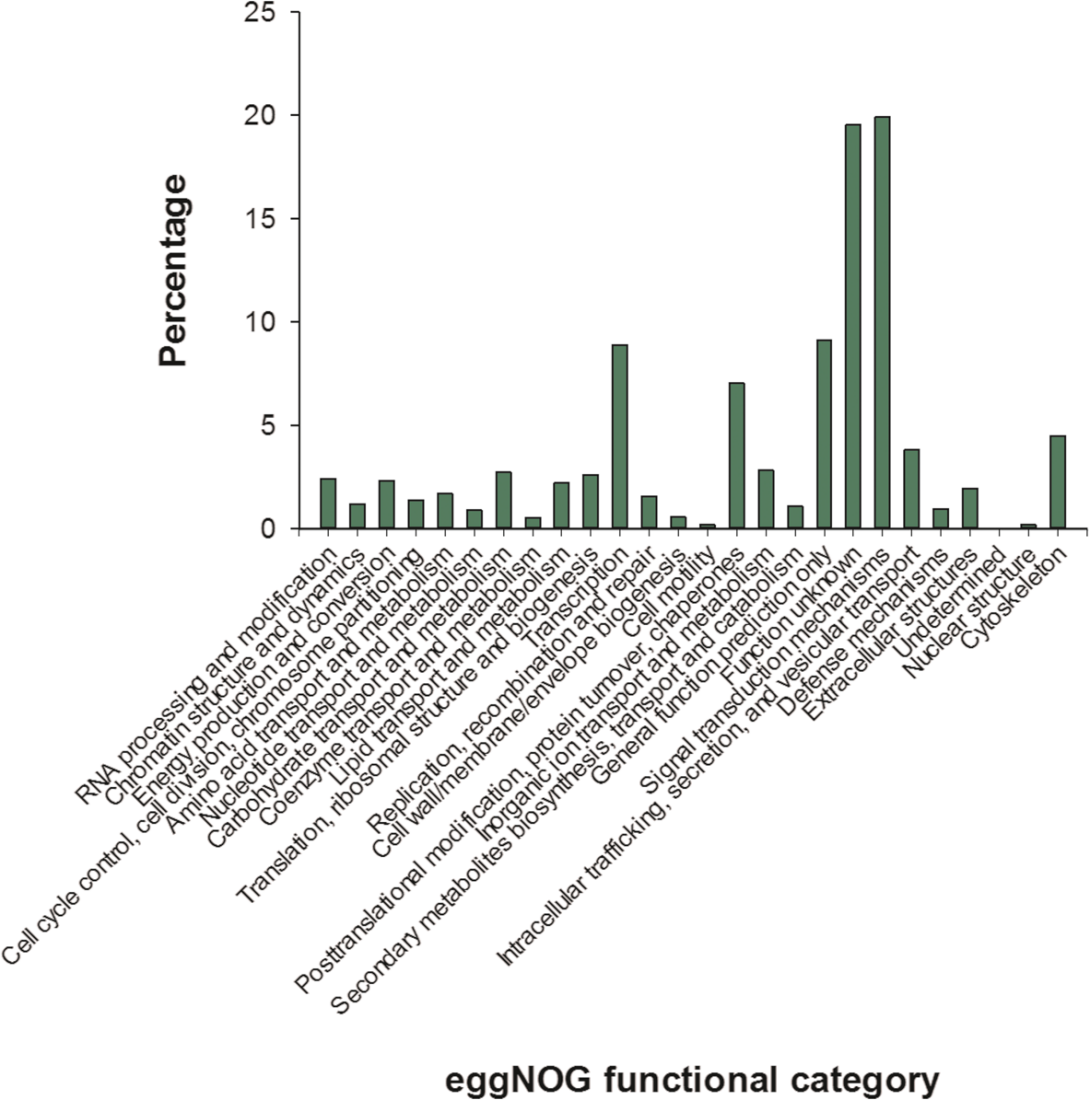
eggNOG function classification of *S. marmoratus* unigenes. A total of 33,291 unigenes were classified into 25 categories

Unigenes were aligned to the eggNOG database for further functional prediction. These data were then classified, and statistical evaluation was conducted for functional predictions of all genes, and the distribution patterns of gene functions of this species were predicted. A total of 31,716 (95.27%) hits were annotated into 33,291 NR top hit unigenes (Table 2) leading to 25 classifications (Fig. 2). Among the functional classes, signal transduction mechanisms (7056, 19.88%) were the largest functional group. The smallest two groups were cell motility (0.17%) and nuclear structure (0.17%). Among these, 330 unigenes were assigned to defense mechanisms (Fig. 2).

**Table 2:** Annotation of unigenes of transcriptomic profiles of *Sebastiscus marmoratus*

Database	Number of annotated unigenes	Percentage of annotated unigenes in NR top hit
Swiss-Prot	29,311	88.04
eggNOG	31,716	95.27
GO	19,698	59.17
KO	11,604	34.86
KEGG	24,202	72.70
NR top hit (Total)	33,291	100

### Identification of GO enrichment analysis and functional classification by KEGG

**Fig. 3:**
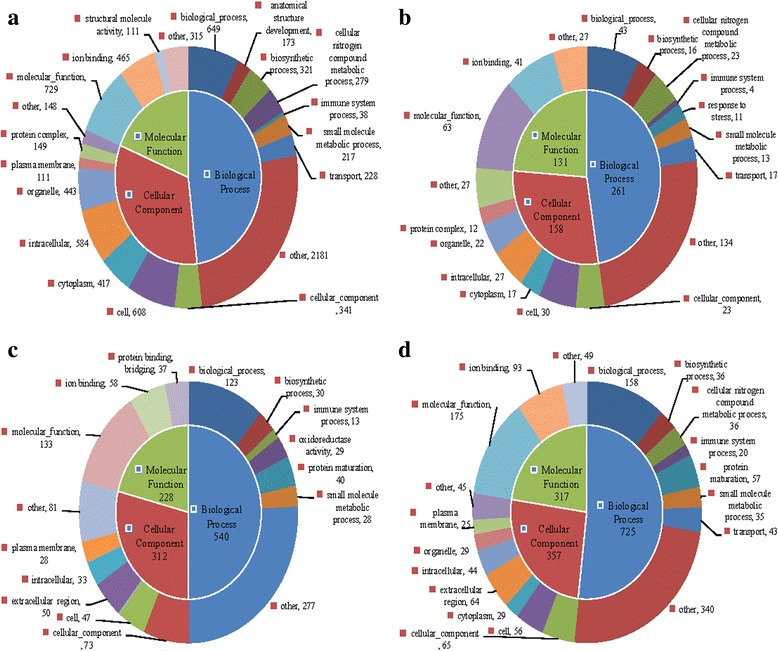
Gene ontology (GO) enrichment analysis of the upregulated genes between *C. irritans*-infected (B, C, D, E) and non-infected (A) *S. marmoratus*, 24 h (group B), 48 h (group C), 72 h (group D) and 96 h (group E) post-challenge and uninfected fish were sampled as control (group A). **a** group B *vs* group A; **b** group C *vs* group A; **c** group D *vs* group A; **d** group E *vs* group A

**Fig. 4:**
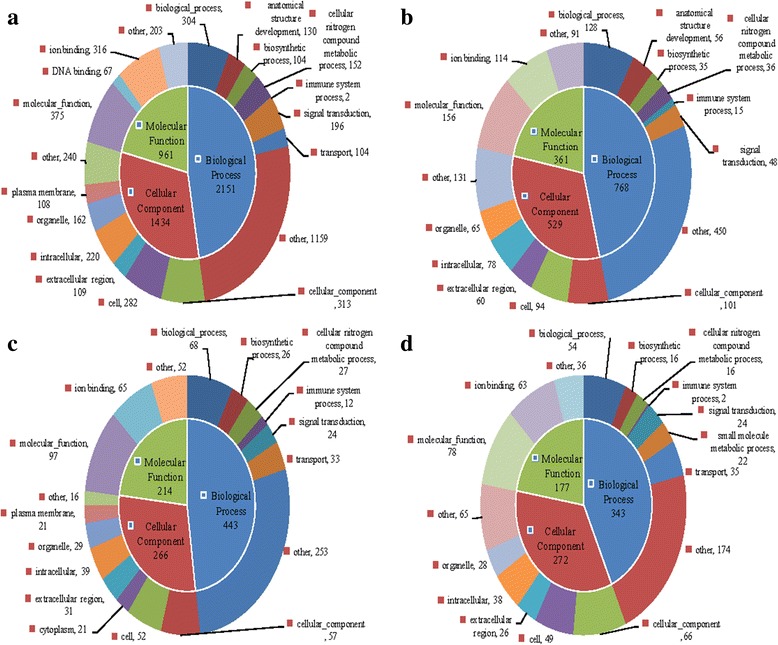
Gene ontology (GO) enrichment analysis of the downregulated genes between *C. irritans*-infected (B, C, D, E) and non-infected (A) *S. marmoratus*, 24 h (group B), 48 h (group C), 72 h (group D) and 96 h (group E) post-challenge and uninfected fish were sampled as control (group A). **a** group B *vs* group A; **b** group C *vs* group A; **c** group D *vs* group A; **d** group E *vs* group A

**Fig. 5:**
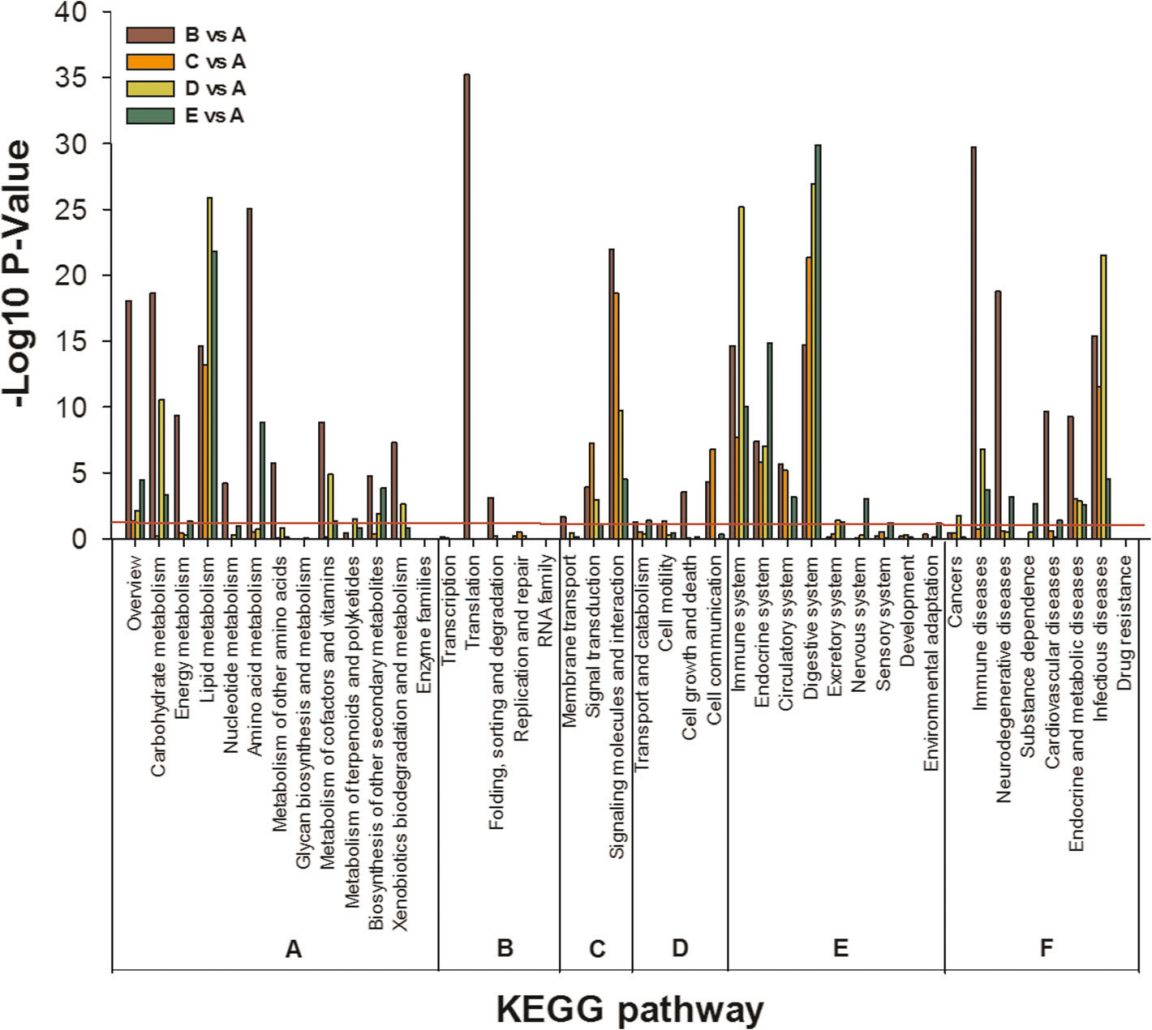
KEGG pathway enrichment analysis of the differently expressed genes between *C. irritans*-infected (B, C, D, E) and non-infected (A) *S. marmoratus*. The red line represents *P* = 0.05. *Key*: A, Metabolism; B, Genetic Information Processing; C, Environmental Information Processing; D, Cellular Processes; E, Organismal Systems; F, Human Diseases

In this study, a total of 19,698 unigenes (59.17%) (Table 2) were assigned predicted GO terms (Figs. 3 and 4). These terms were summarized into 103 sub-categories under 3 GO terms corresponding to the biological process category (73), cellular component category (16), and molecular function category (14). The unigenes were also categorized using the KEGG database to identify the biological pathways in *C. irritans* infected *S. marmoratus*. A total of 24,202 unigenes (72.70%) (Table 2) were further annotated by KEGG and classified into 419 known KEGG pathways (Fig. 5).

To identify the DEGs involved in *S. marmoratus* responded to *C. irritans* infection, pairwise comparisons were carried out between the infection groups (B, C, D and E) and the control group (A) (Figs. 3 and 4). The upregulated DEGs from GO were functionally classified into 94, 68, 72, and 81 sub-categories in groups B, C, D, and E respectively. The downregulated DEGs were classified into 91, 76, 71, and 69 sub-categories in groups B, C, D, and E respectively. The molecular function, biological process, and ion binding genes from *S. marmoratus* infected with *C. irritans* were activated. In a B/A pairwise comparison, 3543 (10.64%) unigenes were differentially expressed, including 1961 upregulated genes in group B. Meanwhile, in C/A, D/A, and E/A pairwise comparisons, only 730, 754, and 814 genes showed differential expression, including 28.36%, 52.79% and 62.78% genes being upregulated, respectively (Fig. 3). In the immune system process sub-categories, there are significant differences between the infection and control groups. In the B/A pairwise comparison, 77 genes showed differential expression, including 38 upregulated IRDEGs (immune-related differentially expressed genes) in group B; meanwhile, in C/A, D/A, and E/A pairwise comparisons, there were only 19, 25 and 22 IRDEGs, including 4, 13 and 20 upregulated genes, respectively (Fig. 3). These results indicated that at 24 hpi, group B is the key time-point for *S. marmoratus* to combat *C. irritans* infection. In the 16 IR-genes in enriched KEGG pathways, the results also indicated that the IR-genes enriched signalling pathways were significantly activated or suppressed in the early stages of *C. irritans* infection. Among these, the most enriched pathways were complement and coagulation cascades, chemokine signalling pathways, and toll-like receptor signalling pathway (Table 3).

**Table 3:** KEGG pathways with differential expressed immune-related genes enrichment between *Cryptocaryon irritans*-infected (B, C, D, and E) and non-infected (A) *Sebastiscus marmoratus*

Pathway ID	Pathway	Genome Unigene number	B *vs* A		C *vs* A		D *vs* A		E *vs* A	
			Up	Down	Up	Down	Up	Down	Up	Down
ko04640	Hematopoietic cell lineage	72	7	10	0	4	5	6	5	3
ko04610	Complement and coagulation cascades	74	37	12	3	5	35	1	36	0
ko04611	Platelet activation	179	5	19	1	13	4	2	5	4
ko04620	Toll-like receptor signalling pathway	106	3	18	0	7	1	10	2	1
ko04621	NOD-like receptor signalling pathway	53	3	2	0	3	0	3	2	0
ko04622	RIG-I-like receptor signalling pathway	69	4	4	0	5	0	6	0	0
ko04623	Cytosolic DNA-sensing pathway	49	3	5	0	1	0	4	1	0
ko04650	Natural killer cell mediated cytotoxicity	86	3	15	1	0	1	1	1	1
ko04612	Antigen processing and presentation	58	9	14	3	0	0	3	2	0
ko04660	T cell receptor signalling pathway	122	1	10	1	0	1	4	0	0
ko04662	B cell receptor signalling pathway	80	1	4	0	0	1	3	0	2
ko04664	Fc epsilon RI signalling pathway	64	0	4	0	2	0	1	0	1
ko04666	Fc gamma R-mediated phagocytosis	114	0	9	0	2	0	2	0	1
ko04670	Leukocyte transendothelial migration	142	7	10	1	4	0	1	3	1
ko04672	Intestinal immune network for IgA production	36	0	11	1	1	0	0	1	0
ko04062	Chemokine signalling pathway	179	5	21	1	8	1	10	4	3
Total		1483	88	168	12	55	49	57	62	17

*Abbreviations: Up* upregulated genes, *Down* downregulated genes

**Fig. 6:**
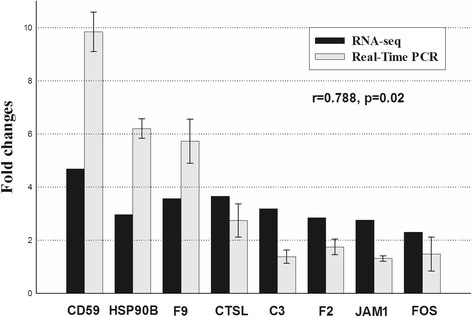
Validation of RNA-seq data by qRT-PCR. The expression of *F9*, *CD59*, *HSP90B*, *JAM1*, *C3*, *F2*, *CTSL*, and *FOS* were detected by RNA-seq (black column) and qRT-PCR (grey column)

To validate the expression profiles of genes identified through Illumina sequencing, the relative mRNA levels of the following eight innate immune-relevant genes (*F9*, *CD59*, *HSP90B*, *JAM1*, *C3*, *F2*, *CTSL*, and *FOS*) were analyzed by qRT-PCR (Fig. 6). As presented in Fig. 6, the qRT-PCR results correlated well with the results obtained through RNA-Seq.

### Analysis of immune-related signalling pathways in infected fish

Transcriptomic studies of the *S. marmoratus* immune responses to *C. irritans* infection have been conducted. To our knowledge, all of them focus on immune variation at a single timepoint post-infection [6, 22, 23]. It is well established that during the development of *C. irritans* cells, the trophont grows up from 20 μm to 300 μm. Four days post-infection, a large number of trophonts start to leave the host after maturation [33]. However, the changes of immune responses of *S. marmoratus* to *C. irritans* trophonts at different developmental stages remain largely unknown. Most of the current studies focus on mono-molecular or mono-pathway variations post*-C. irritans* infection with the passage of time [17]. In this study, we obtained the most comprehensive data to date through the analysis and bioinformatical enrichment of immune-related signalling pathways, including complement and coagulation cascades, chemokine signalling pathway, and toll-like receptor signalling pathway as previously reported [6, 22, 23]. What’s new is that the analysis of transcriptomic variation with the passage of time revealed the variation in immune responses and the key time-point for the fish to cope with the *C. irritans* infection.

#### Toll-like receptor signalling pathway

**Fig. 7:**
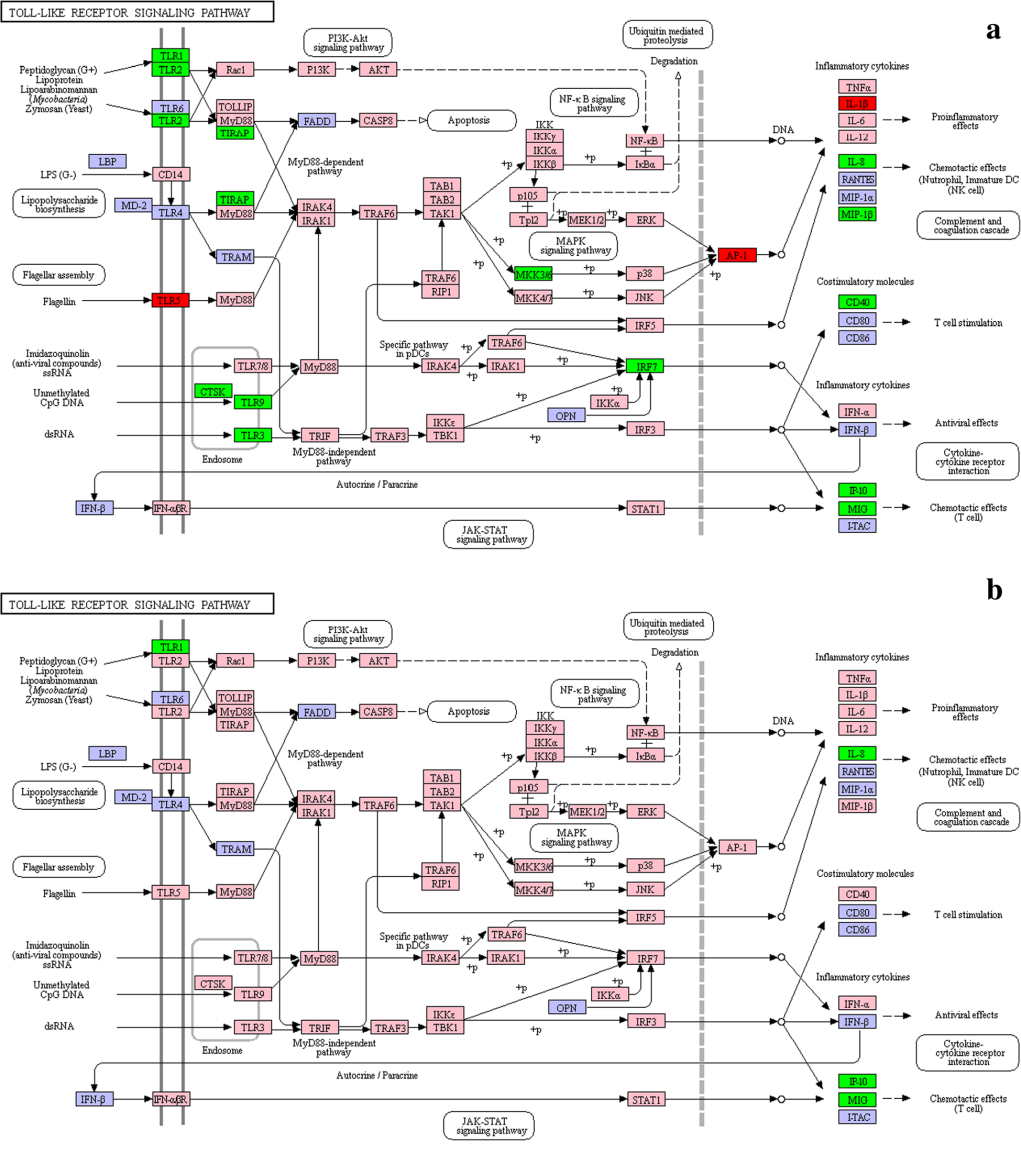
List of genes involved in Toll-like receptor pathway generated by KEGG of the differently expressed genes between *Cryptocaryon irritans*-infected and non-infected *S. marmoratus*, 24 h (group B) and 48 h (group C) post-challenge and uninfected fish were sampled as control (group A). **a** group B *vs* group A; **b** group C *vs* group A. Red indicates significantly increased expression; green indicates significantly decreased expression, and pink indicates unchanged expression. Blue denotes genes that were not identified in the expression profile analysis

**Fig. 8:**
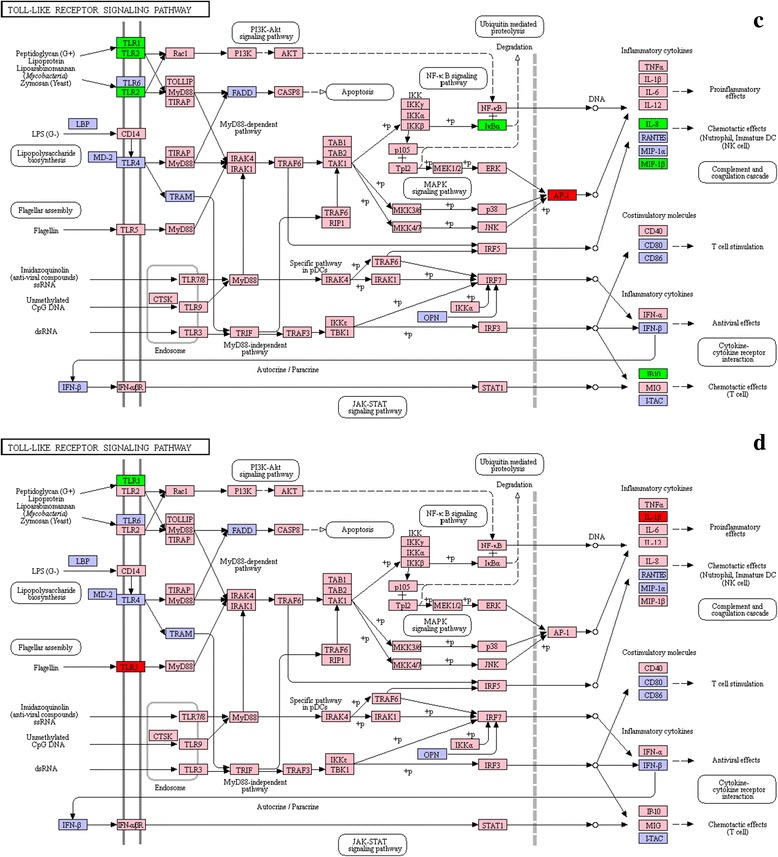
List of genes involved in Toll-like receptor pathway generated by KEGG of the differently expressed genes between *Cryptocaryon irritans*-infected and non-infected *S. marmoratus*, 72 h (group D) and 96 h (group E) post-challenge and uninfected fish were sampled as control (group A). **c** group D *vs* group A; **d** group E *vs* group A. Red indicates significantly increased expression; green indicates significantly decreased expression, and pink indicates unchanged expression. Blue denotes genes that were not identified in the expression profile analysis

Innate immunity acts as the first line of immune system defence against infection by pattern-recognition receptors (PRRs). Toll-like receptors, a family of type I transmembrane proteins, are one of best characterized PRRs. It is well established that toll-like receptors recognize conserved pathogen-associated molecular patterns (PAMPs) such as peptidoglycan (PGN), lipopolysaccharide (LPS), lipoprotein (LP), flagellin, and viral dsRNA. Activation of toll-like receptors by the corresponding PAMPs initiates signalling cascades leading to the activation of transcription factors, such as NF-κB, AP-1 and interferon regulatory factors (IRFs). Through a series of signalling cascades, toll-like receptors can activate various cellular responses, including the production of interferons (IFNs), pro-inflammatory cytokines and effector cytokines. Research on *C. irritans* infected *E. coioides* indicated that *TLR9* and *TLR21* transcripts were induced in skin and gill [34]; *TLR2* was upregulated in the head kidney and spleen 6 h post-infection but was downregulated in the skin and gill at most of the tested time points. Furthermore, *MyD88*, TRAF6, and *IL1β* were upregulated in immune tissues at most time points [10, 14]. In addition, a transcriptome sequencing on *C. irritans* infected *L. crocea* showed that *TLR5* might be involved in identifying the *C. irritans*’ antigen composition, which was corroborated by the finding of Bai et al. (2017), indicating that *C. irritans* infection could significantly upregulate the expression of *EcTLR5s* in the gill and spleen [35]. Additionally, it has been shown that the *MyD88* gene plays a key role in resistance to *C. irritans.* Upregulated *IKKb*, *AP-1 (JUN, FOS)*, *IRF3*, *IRF7* and *STAT1* promoted expression of pro-inflammatory and inflammatory cytokines, such as *TNFa*, *IL1β*, *IL6*, *IFNa*, and *IFNβ*. In this study, *TLR5*, *FOS*, and *IL1β* were upregulated 24 h after *C. irritans* infection (Fig. 7, Additional file 4: Table S4). Specifically, *FOS* was upregulated at 72 hpi, and *TLR5* and *IL1β* were also upregulated at 96 hpi (Fig. 8, Additional file 4: Table S4). Previous data indicated that in bacterial infection, *TLR5* sensing occurs via recognition of a variety of bacterial flagellins serves to augment the activation of NF-κB [36, 37]. However, this study verified that *TLR5* was the only activated receptor, *AP-1 (FOS)* was the key transcription factor, and *IL1β* functioning as a pro-inflammatory cytokine was promoted by *C. irritans* infection. To date, flagellin and profilin [38] are the only known ligands for *TLR5*. However, whether *SmTLR5* could mediate *IL1β* production in response to *C. irritans* profilin (https://www.ncbi.nlm.nih.gov/nuccore/449138907) or which *C. irritans* ligands had been recognized by *SmTLR5* is unknown. Interestingly, expression of *TLR1*, *TLR2*, *TLR3*, *TLR8*, *TLR9*, *CTSK*, *TIRAP*, *MAP2K6*, *IRF7*, *IL12β*, *IL8*, *CCL4, TNFRSF5*, *CXCL10*, and *CXCL9* were significantly downregulated 24 h after infection with *C. irritans* in this study (Fig. 7, Additional file 4: Table S4). A current report has indicated that transcription of *TLR21* and *TLR9* was downregulated in spleen and head kidney, suggesting that these TLR genes play a role in host anti-*C. irritans* immune responses [34]. Alternatively, it was suggested that the downregulated expression of the TLR genes-*TAK1, PIK3C* and *PIK3R* might be involved in the negative regulation mechanism for an over-inflammatory response following *C. irritans* infection [22].

#### Cytokine-cytokine receptor interaction and chemokine signalling pathway

Interleukins (IL) play an important role in the regulation of the immune system during infections [39]. In this study, an upregulation of *IL1β, IL1R2* and *IL11* were observable at 24 hpi (Additional file 4: Table S4). Current work demonstrated that a significant upregulation of *IL1β* indicated that *I. multifiliis* and *C. irritans* infection elicited an inflammatory response in the host [10, 40]. The biological effects of *IL1β* are mediated through interactions with *IL1R1* [41, 42], whereas the *IL1R2* acts as a decoy for *IL1β* inhibiting its activity [43]. In the skin and head kidney of rainbow trout (*Oncorhynchus mykiss*) infected with *I. multifiliis*, transcription of *IL1R2* was significantly upregulated [40]. This suggests that *IL1R2* may play a role in the prevention of *IL1β* entering the systemic circulation from the sites of inflammation [44]. In the cytokine network, *IL11* either by itself or in synergy with other growth factors stimulates the proliferation and differentiation of both early and late hematopoietic progenitors [45, 46]. Podok et al. [47] reported upregulation of *IL11* expression 6 or 72 hpi in Crucian carp (*Carassius auratus gibelio*) was induced by *Aeromonas hydrophila*. In this study, we believe that the upregulation of the *IL11* gene might play a role in the immune response to *C. irritans* infection.

Chemokines are a group of small molecules, which regulate the trafficking of different types of leukocytes between cells and thereby playing an important role in the functioning of the immune system as well as homeostasis and development [48]. In mammals, chemokines are classified into four main subfamilies, including CXC, CC, CX3C, and XC [49]. In a transcriptome study of the Japanese flounder (*Paralichthys olivaceus*) spleen, 20 chemokines have been identified, which correspond to CC and CXC chemokines and chemokine receptors [29]. In the *L. crocea* liver transcriptome, the significantly upregulated *CXCL10/12* and *CCL19/20/25* genes might play a crucial role in the immune response to *C. irritans* [22]. In this study, 179 CSP related genes were identified in *S. marmoratus* systemic tissues; including 7 CXC (*CXCL5/6/8/9/10/12/13/14*), 4 CXC receptors (*CXCR2/3/4/5*), 4 CC (*CCL4/19/20/25*), 6 CC receptors (*CCR3/4/5/6/7/9*) and 1 XC receptor (*XCR1*) were identified for the first time in *S. marmoratus.* However, only 5 in 26, 1 in 9, 1 in 11 and 4 in 7 CSP related DEGs were significantly upregulated at the 24 h (group B/A), 48 h (group C/A), 72 h (group D/A), and 96 h (group E/A), post-*C. irritans* infection, respectively (Additional file 4: Table S4). Additionally, fourteen chemokine signalling pathway related DEGs were identified, among which, *CCL1*, *CCL4* and *CXCL9–11* were upregulated in the skin locally infected sites, suggesting teleost skin-associated lymphoid tissue recruits different immune cells, activating a unique immune response pattern [23]. Furthermore, it has been speculated that the downregulated CSP related genes might be involved in the negative regulatory mechanism of the inflammatory response following *C. irritans* infection [22].

#### Complement cascade signalling pathway

**Fig. 9:**
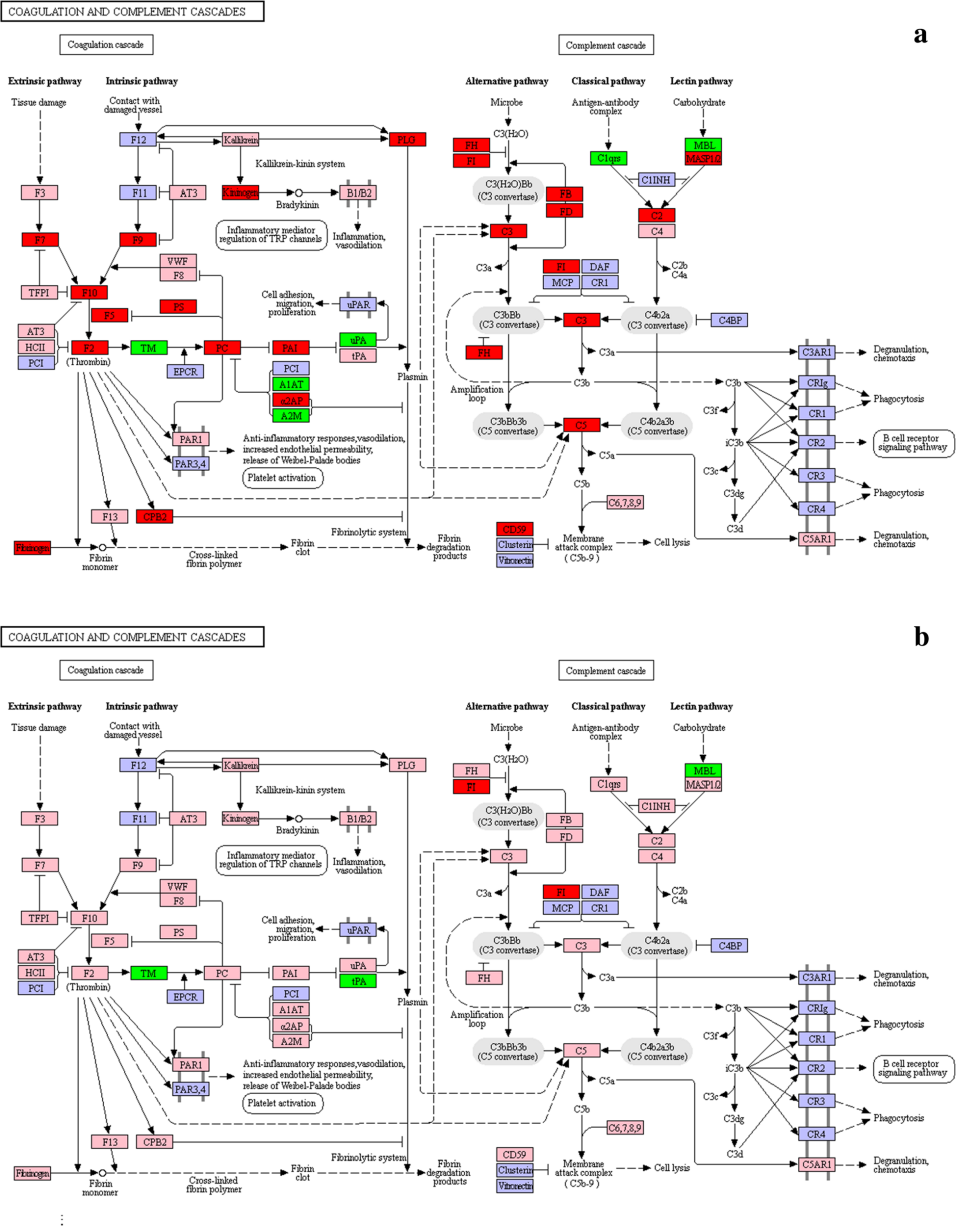
List of genes involved in the complement and coagulation cascades pathway generated by KEGG of the differently expressed genes between *C. irritans*-infected and non-infected *S. marmoratus*, 24 h (group B) and 48 h (group C) post-challenge and uninfected fish were sampled as control (group A). **a** group B *vs* group A; **b** group C *vs* group A. Red indicates significantly increased expression; green indicates significantly decreased expression; and pink indicates unchanged expression. Blue denotes genes that were not identified in the expression profile analysis

**Fig. 10:**
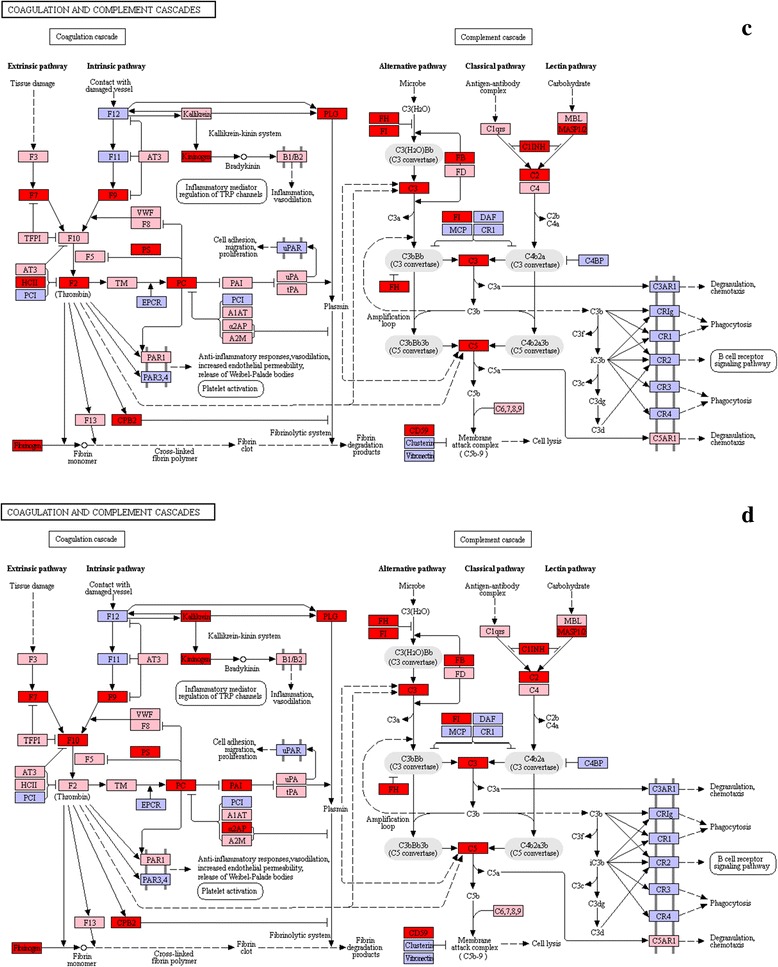
List of genes involved in the complement and coagulation cascades pathway generated by KEGG of the differently expressed genes between *C. irritans*-infected and non-infected *S. marmoratus*, 72 h (group D) and 96 h (group E) post-challenge and uninfected fish were sampled as control (group A). **c** group D *vs* group A; **d** group E *vs* group A. Red indicates significantly increased expression; green indicates significantly decreased expression; and pink indicates unchanged expression. Blue denotes genes that were not identified in the expression profile analysis

Studies showed that, in response to *C. irritans* infection, the fish body mainly activated its complement system via the alternative pathway (AP) [6, 12]. This was shown to be largely involved in non-specific immunity during the early stage of infection [12, 21]. When no antigen-antibody complex was formed or *C1*, *C4* or *C2* was absent in the body, the invading pathogenic substances would directly activate *C3*, ultimately *C5*-*C9* [50, 51]. In a recent study of *I. multifiliis* infected rainbow trout (*Oncorhynchus mykiss*), *C3* expression increased by as much as 5.3-fold at days 4 and 6 in the skin, and at days 6 and 26 in the spleen [52]. From the *L. crocea* liver transcriptome, Wang et al. only identified *FH* and *C3*-like genes, which belongs to the alternative pathway activation. *FH* and *C3*-like expression were upregulated through experimental infection with *C. irritans* [22]. In this study, the AP-related genes *FI*, *FH*, *FD*, *FB*, *C3*, *C5*, *C7*, *C8A*, *C8B*, *C8G* and *C9* were significantly upregulated (Figs. 9 and 10, Additional file 4: Table S4). *C1* complex-*C1qrs* is the initiation factor for the classical pathway, while *MASP1/2* and *MBL* are the factors known to activate the lectin pathway. The data presented here indicate that, although the expression of *C2* and *MASP1/2* were upregulated, *C1qrs* and *MBL* were downregulated, probably because of the inhibition by *C1INH*. This could explain why *C4* was not activated. However, the membrane-bound complement regulatory protein, *CD59*, which acts to limit the assembly of membrane attack complex (MAC), was significantly upregulated, to prevent the body’s tissue cells from being accidentally damaged by the complement system [53, 54]. On this basis, the results of this study verified the role of the *S. marmoratus* complement alternative pathway in response to *C. irritans* infection [6].

## Conclusions

Infection with *C. irritans* caused a large number of DEGs in the IR-tissues of *S. marmoratus*. However, with the passage of time after infection, the number of DEGs was significantly reduced from 24 h to 48 h; then increased again from 72 h to 96 h. Specifically, the IRDEGs, which belong to the pathways such as complement cascades, cytokine-cytokine receptor interaction, chemokine signalling pathways, and toll-like receptor signalling pathway were mainly found at early signalling following.

## Additional files

Additional file 1: Table S1. The dispersion factor of all of the unigenes between treatment and control groups. (XLSX 3827 kb)

Additional file 2: Table S2. Primers for eight target immune-relevant genes used for qRT-PCR. (DOCX 14 kb)

Additional file 3: Table S3. *S. marmoratus* transcriptome expression profile after *C. irritans* infection. (DOCX 16 kb)

Additional file 4: Table S4. Immune systems with differentially expressed genes between *C. irritans*-infected (B, C, D, E) and non-infected (A) *S. marmoratus. (DOCX 133 kb)*

## Abbreviations

DEGs: differentially expressed genes
eggNOG: evolutionary genealogy of genes: non-supervised orthologous groups
GO: gene ontology
IRDEGs: immune-related differentially expressed genes
KEGG: kyoto encyclopedia of genes and genomes
qRT-PCR: quantitative real-time polymerase chain reaction
RNA-seq: RNA sequencing
