# Supplementary material for: Transcriptomic analysis reveals the key immune-related signalling pathways of Sebastiscus marmoratus in response to infection with the parasitic ciliate Cryptocaryon irritans
Source: Parasit Vectors. 2017 Nov 21;10:576. doi: 10.1186/s13071-017-2508-7 (PMC5697091; doi:10.1186/s13071-017-2508-7)
Supplement: Supplementary file 2 — Primers for eight target immune-relevant genes used for qRT-PCR. (DOCX 14 kb) [file 13071_2017_2508_MOESM2_ESM.docx]

**Table S2** Primers for eight target immune-relevant genes used for qRT-PCR.

| Primer | Primer sequence (5’-3’) |
| --- | --- |
| F9-F | CTTAAACGCCCTGCACACAC |
| F9-R | ACTCGGGAGGATGTCACTCA |
| C3-F | CCTCGCAGGATGTTTGGTCT |
| C3-R | TACCAGAAGCCGTGTTGGAC |
| CD59-F | ACCGAGACAGACGAATCAGC |
| CD59-R | GCCATTTTGGAGACACACCG |
| HSP90B-F | AGGTCCATCCTGTTTGTGCC |
| HSP90B-R | ATGTCGTTGAAGTCGTCGGT |
| JAM1-F | AGAGCCACTATTACGCCAGC |
| JAM1-R | TTCAACGATGCTGGTCCTCC |
| F2-F | GTCGCCGATGTAGTCCACTC |
| F2-R | GCAGGAGAACTTCTGTCGGA |
| CTSL-F | GGACCCGTTTCAGTCGCTAT |
| CTSL-R | TCGAAGCCATAACCCACCAC |
| FOS-F | GGTGCCAACCCCTGTATGTT |
| FOS-R | ATGTGGTGGAGGTAGGGACA |
| β-actin-4F | AGGGAAATCGTGCGTGAC |
| β-actin-4R | TGATGCTGTTGTAGGTGGTCT |
