# Supplementary material for: Transcriptomic analysis reveals the key immune-related signalling pathways of Sebastiscus marmoratus in response to infection with the parasitic ciliate Cryptocaryon irritans
Source: Parasit Vectors. 2017 Nov 21;10:576. doi: 10.1186/s13071-017-2508-7 (PMC5697091; doi:10.1186/s13071-017-2508-7)
Supplement: Supplementary file 3 — S. marmoratus transcriptome expression profile after C. irritans infection. (DOCX 16 kb) [file 13071_2017_2508_MOESM3_ESM.docx]

**Table S3 *S. marmoratus* transcriptome expression profile after *C. irritans* infection**

| Statistics of raw data | | | | | |
| --- | --- | --- | --- | --- | --- |
| Sample | Reads | Raw Reads | Raw Data(bp) | Reads Len.(bp) | |
| A | R1 | 24,533,456 | 2,477,879,056 | 101 | |
|  | R2 | 24,533,456 | 2,477,879,056 |  |  |
|  | Paired | 24,533,456 | 4,955,758,112 |  |  |
| B | R1 | 36,628,922 | 3,699,521,122 |  |  |
|  | R2 | 36,628,922 | 3,699,521,122 |  |  |
|  | Paired | 36,628,922 | 7,399,042,244 |  |  |
| C | R1 | 36,845,002 | 3,721,345,202 |  |  |
|  | R2 | 36,845,002 | 3,721,345,202 |  |  |
|  | Paired | 36,845,002 | 7,442,690,404 |  |  |
| D | R1 | 32,712,799 | 3,303,992,699 |  |  |
|  | R2 | 32,712,799 | 3,303,992,699 |  |  |
|  | Paired | 32,712,799 | 6,607,985,398 |  |  |
| E | R1 | 34,722,123 | 3,506,934,423 |  |  |
|  | R2 | 34,722,123 | 3,506,934,423 |  |  |
|  | Paired | 34,722,123 | 7,013,868,846 |  |  |
|  |  |  |  |  |  |
| Filtered Statistics (Q20) | | | | | |
| Sample | Reads | Clean Reads | Clean Data(bp) | Useful Reads % | Useful Data % |
| A | R1 | 22,125,687 | 2,202,916,369 | 90.19% | 88.57% |
|  | R2 | 22,125,687 | 2,186,299,487 |  |  |
|  | Paired | 22,125,687 | 4,389,215,856 |  |  |
| B | R1 | 33,263,795 | 3,312,589,643 | 90.81% | 89.25% |
|  | R2 | 33,263,795 | 3,290,914,036 |  |  |
|  | Paired | 33,263,795 | 6,603,503,679 |  |  |
| C | R1 | 33,316,906 | 3,316,042,036 | 90.42% | 88.82% |
|  | R2 | 33,316,906 | 3,294,567,507 |  |  |
|  | Paired | 33,316,906 | 6,610,609,543 |  |  |
| D | R1 | 29,760,020 | 2,962,722,649 | 90.97% | 89.40% |
|  | R2 | 29,760,020 | 2,944,945,117 |  |  |
|  | Paired | 29,760,020 | 5,907,667,766 |  |  |
| E | R1 | 31,516,989 | 3,136,872,723 | 90.77% | 89.17% |
|  | R2 | 31,516,989 | 3,117,427,579 |  |  |
|  | Paired | 31,516,989 | 6,254,300,302 |  |  |
