# Supplementary material for: Transcriptomic analysis reveals the key immune-related signalling pathways of Sebastiscus marmoratus in response to infection with the parasitic ciliate Cryptocaryon irritans
Source: Parasit Vectors. 2017 Nov 21;10:576. doi: 10.1186/s13071-017-2508-7 (PMC5697091; doi:10.1186/s13071-017-2508-7)
Supplement: Supplementary file 4 — Immune systems with differentially expressed genes between C. irritans-infected (B, C, D, E) and non-infected (A) S. marmoratus. (DOCX 133 kb) [file 13071_2017_2508_MOESM4_ESM.docx]

**Table S4 Immune systems with differentlly expressed genes between *C. irritans*-infected (B, C, D, E) and non-infected (A) *S. marmoratus***

| **B vs A** |  |  | **Unigene ID** | | **Description** | | | | |  | **Fold**  **Change** | **log_2_ Fold Change** | **P Value** |
| --- | --- | --- | --- | --- | --- | --- | --- | --- | --- | --- | --- | --- | --- |
| **ko04640** | **Hematopoietic cell lineage** | | | | | | | | |  |  |  |  |
|  |  | ko:K01389 | comp54819_c0_seq2 | | MME; neprilysin [EC:3.4.24.11] | | | | | Down | 0.37 | -1.45 | 0.0169 |
|  |  | ko:K04008 | comp54684_c0_seq1 | | CD59; CD59 antigen | | | | | Up | 4.68 | 2.23 | 0.0001 |
|  |  | ko:K04008 | comp57062_c0_seq1 | | CD59; CD59 antigen | | | | | Up | 5.83 | 2.54 | 0.0000 |
|  |  | ko:K04387 | comp59281_c0_seq2 | | IL1R2; interleukin 1 receptor type II | | | | | Up | 84.84 | 6.41 | 0.0000 |
|  |  | ko:K04519 | comp24971_c0_seq1 | | IL1B; interleukin 1 beta | | | | | Up | 5.12 | 2.36 | 0.0005 |
|  |  | ko:K05072 | comp61812_c0_seq7 | | IL7R; interleukin 7 receptor | | | | | Down | 0.28 | -1.86 | 0.0028 |
|  |  | ko:K05090 | comp63551_c1_seq3 | | CSF1R, FMS; macrophage colony-stimulating factor 1 receptor [EC:2.7.10.1] | | | | | Down | 0.34 | -1.56 | 0.0039 |
|  |  | ko:K05092 | comp71006_c0_seq1 | | FLT3, FLK2; fms-related tyrosine kinase 3 [EC:2.7.10.1] | | | | | Down | 0.42 | -1.26 | 0.0178 |
|  |  | ko:K05417 | comp59437_c0_seq4 | | IL11; interleukin 11 | | | | | Up | 6.67 | 2.74 | 0.0001 |
|  |  | ko:K05423 | comp60827_c2_seq1 | | CSF3, GCSF; granulocyte colony-stimulating factor | | | | | Up | 6.28 | 2.65 | 0.0016 |
|  |  | ko:K06449 | comp68241_c0_seq10 | | CD2; CD2 antigen | | | | | Down | 0.43 | -1.21 | 0.0454 |
|  |  | ko:K06451 | comp68592_c0_seq1 | | CD3E; T-cell surface glycoprotein CD3 epsilon chain | | | | | Down | 0.25 | -1.99 | 0.0017 |
|  |  | ko:K06451 | comp68592_c0_seq3 | | CD3E; T-cell surface glycoprotein CD3 epsilon chain | | | | | Down | 0.33 | -1.61 | 0.0110 |
|  |  | ko:K06458 | comp68785_c0_seq8 | | CD8A; CD8A antigen, alpha polypeptide | | | | | Down | 0.25 | -2.01 | 0.0009 |
|  |  | ko:K06467 | comp70355_c2_seq3 | | CD22, SIGLEC2; CD22 antigen | | | | | Down | 0.28 | -1.83 | 0.0011 |
|  |  | ko:K06480 | comp71078_c8_seq1 | | ITGA1; integrin alpha 1 | | | | | Down | 0.30 | -1.76 | 0.0032 |
|  |  | ko:K06503 | comp64406_c3_seq4 | | TFRC, CD71; transferrin receptor | | | | | Up | 0.34 | -1.54 | 0.0068 |
| **ko04610** | **Complement and coagulation cascades** | | | | | | | | |  |  |  |  |
|  |  | ko:K01300 | comp65823_c0_seq1 | | CPB2; carboxypeptidase B2 [EC:3.4.17.20] | | | | | Up | 2.15 | 1.11 | 0.0396 |
|  |  | ko:K01313 | comp32288_c0_seq1 | | F2; coagulation factor II (thrombin) [EC:3.4.21.5] | | | | | Up | 2.84 | 1.50 | 0.0044 |
|  |  | ko:K01314 | comp55633_c0_seq1 | | F10; coagulation factor X [EC:3.4.21.6] | | | | | Down | 0.30 | -1.74 | 0.0035 |
|  |  | ko:K01314 | comp45692_c0_seq2 | | F10; coagulation factor X [EC:3.4.21.6] | | | | | Up | 2.09 | 1.07 | 0.0433 |
|  |  | ko:K01315 | comp58611_c0_seq1 | | PLG; plasminogen [EC:3.4.21.7] | | | | | Up | 2.39 | 1.26 | 0.0167 |
|  |  | ko:K01320 | comp43168_c0_seq1 | | F7; coagulation factor VII [EC:3.4.21.21] | | | | | Up | 2.41 | 1.27 | 0.0183 |
|  |  | ko:K01320 | comp54518_c0_seq1 | | F7; coagulation factor VII [EC:3.4.21.21] | | | | | Up | 5.04 | 2.33 | 0.0000 |
|  |  | ko:K01320 | comp57677_c0_seq1 | | F7; coagulation factor VII [EC:3.4.21.21] | | | | | Up | 2.50 | 1.32 | 0.0126 |
|  |  | ko:K01321 | comp62328_c0_seq2 | | F9; coagulation factor IX (Christmas factor) [EC:3.4.21.22] | | | | | Up | 7.75 | 2.95 | 0.0000 |
|  |  | ko:K01321 | comp64057_c0_seq1 | | F9; coagulation factor IX (Christmas factor) [EC:3.4.21.22] | | | | | Up | 3.57 | 1.84 | 0.0007 |
|  |  | ko:K01330 | comp58616_c0_seq2 | | C1R; complement component 1, r subcomponent [EC:3.4.21.41] | | | | | Up | 2.32 | 1.21 | 0.0210 |
|  |  | ko:K01331 | comp58592_c0_seq1 | | C1S; complement component 1, s subcomponent [EC:3.4.21.42] | | | | | Down | 0.48 | -1.05 | 0.0468 |
|  |  | ko:K01332 | comp67844_c1_seq9 | | C2; complement component 2 [EC:3.4.21.43] | | | | | Up | 2.18 | 1.12 | 0.0334 |
|  |  | ko:K01333 | comp64792_c0_seq2 | | CFI; complement factor I [EC:3.4.21.45] | | | | | Up | 10.51 | 3.39 | 0.0000 |
|  |  | ko:K01334 | comp58630_c0_seq1 | | CFD; component factor D [EC:3.4.21.46] | | | | | Up | 2.83 | 1.50 | 0.0046 |
|  |  | ko:K01335 | comp14093_c0_seq1 | | CFB; component factor B [EC:3.4.21.47] | | | | | Up | 3.79 | 1.92 | 0.0003 |
|  |  | ko:K01344 | comp51226_c0_seq1 | | PROC; protein C (activated) [EC:3.4.21.69] | | | | | Up | 3.26 | 1.70 | 0.0016 |
|  |  | ko:K01348 | comp65895_c0_seq3 | | PLAU; urokinase plasminogen activator [EC:3.4.21.73] | | | | | Down | 0.37 | -1.45 | 0.0378 |
|  |  | ko:K03898 | comp65879_c0_seq1 | | KNG; kininogen | | | | | Up | 3.13 | 1.64 | 0.0020 |
|  |  | ko:K03902 | comp70182_c0_seq1 | | F5; coagulation factor V (labile factor) | | | | | Up | 2.07 | 1.05 | 0.0470 |
|  |  | ko:K03903 | comp63621_c1_seq6 | | FGA; fibrinogen alpha chain | | | | | Up | 2.42 | 1.27 | 0.0154 |
|  |  | ko:K03904 | comp54730_c0_seq1 | | FGB; fibrinogen beta chain | | | | | Up | 2.33 | 1.22 | 0.0197 |
|  |  | ko:K03905 | comp40601_c0_seq1 | | FGG; fibrinogen gamma chain | | | | | Up | 3.94 | 1.98 | 0.0002 |
|  |  | ko:K03907 | comp66488_c1_seq7 | | THBD, CD141; thrombomodulin | | | | | Down | 0.41 | -1.30 | 0.0274 |
|  |  | ko:K03908 | comp70226_c1_seq1 | | PROS1; protein S | | | | | Up | 2.33 | 1.22 | 0.0227 |
|  |  | ko:K03910 | comp69215_c0_seq2 | | A2M; alpha-2-macroglobulin | | | | | Down | 0.25 | -2.00 | 0.0002 |
|  |  | ko:K03910 | comp69471_c2_seq3 | | A2M; alpha-2-macroglobulin | | | | | Down | 0.40 | -1.33 | 0.0120 |
|  |  | ko:K03982 | comp65845_c0_seq1 | | SERPINE1, PAI1; plasminogen activator inhibitor-1 | | | | | Up | 0.17 | -2.53 | 0.0005 |
|  |  | ko:K03983 | comp63530_c1_seq1 | | SERPINF2, AAP; alpha-2-antiplasmin | | | | | Up | 2.23 | 1.16 | 0.0287 |
|  |  | ko:K03984 | comp62255_c0_seq2 | | SERPINA1, AAT; alpha-1-antitrypsin | | | | | Down | 0.36 | -1.46 | 0.0057 |
|  |  | ko:K03987 | comp47537_c0_seq1 | | C1QB; complement C1q subcomponent subunit B | | | | | Down | 0.44 | -1.18 | 0.0251 |
|  |  | ko:K03988 | comp14044_c0_seq1 | | C1QG; complement C1q subcomponent subunit C | | | | | Down | 0.46 | -1.12 | 0.0320 |
|  |  | ko:K03988 | comp71848_c0_seq1 | | C1QG; complement C1q subcomponent subunit C | | | | | Down | 0.45 | -1.17 | 0.0262 |
|  |  | ko:K03990 | comp57282_c0_seq1 | | C3; complement component 3 | | | | | Up | 0.24 | -2.08 | 0.0002 |
|  |  | ko:K03991 | comp58993_c0_seq3 | | MBL; mannose-binding lectin | | | | | Down | 0.19 | -2.40 | 0.0001 |
|  |  | ko:K03992 | comp65138_c2_seq2 | | MASP1; mannan-binding lectin serine protease 1 [EC:3.4.21.-] | | | | | Up | 5.31 | 2.41 | 0.0068 |
|  |  | ko:K03992 | comp65138_c2_seq5 | | MASP1; mannan-binding lectin serine protease 1 [EC:3.4.21.-] | | | | | Up | 8.49 | 3.09 | 0.0002 |
|  |  | ko:K03994 | comp54789_c0_seq1 | | C5; complement component 5 | | | | | Up | 3.30 | 1.72 | 0.0012 |
|  |  | ko:K03996 | comp64828_c0_seq1 | | C7; complement component 7 | | | | | Down | 0.34 | -1.57 | 0.0076 |
|  |  | ko:K03996 | comp71185_c1_seq1 | | C7; complement component 7 | | | | | Up | 14.53 | 3.86 | 0.0000 |
|  |  | ko:K03997 | comp65864_c0_seq1 | | C8A; complement component 8 subunit alpha | | | | | Up | 4.54 | 2.18 | 0.0001 |
|  |  | ko:K03998 | comp62206_c0_seq1 | | C8B; complement component 8 subunit beta | | | | | Up | 6.13 | 2.62 | 0.0000 |
|  |  | ko:K03999 | comp60438_c0_seq1 | | C8G; complement component 8 subunit gamma | | | | | Up | 6.67 | 2.74 | 0.0000 |
|  |  | ko:K04000 | comp66790_c0_seq2 | | C9; complement component 9 | | | | | Up | 18.18 | 4.18 | 0.0000 |
|  |  | ko:K04001 | comp61753_c0_seq1 | | SERPING1, C1INH; C1 inhibitor | | | | | Up | 2.26 | 1.17 | 0.0252 |
|  |  | ko:K04004 | comp67044_c0_seq7 | | HF1; complement factor H | | | | | Up | 11.60 | 3.54 | 0.0000 |
|  |  | ko:K04004 | comp68996_c0_seq3 | | HF1; complement factor H | | | | | Up | 3.49 | 1.80 | 0.0007 |
|  |  | ko:K04008 | comp54684_c0_seq1 | | CD59; CD59 antigen | | | | | Up | 4.68 | 2.23 | 0.0001 |
|  |  | ko:K04008 | comp57062_c0_seq1 | | CD59; CD59 antigen | | | | | Up | 5.83 | 2.54 | 0.0000 |
| **ko04611** | **Platelet activation** | | | |  | | | | |  |  |  |  |
|  |  | ko:K00509 | comp66715_c0_seq3 | | PTGS1, COX1; prostaglandin-endoperoxide synthase 1 [EC:1.14.99.1] | | | | | Down | 0.26 | -1.92 | 0.0116 |
|  |  | ko:K00907 | comp29446_c0_seq1 | | MYLK; myosin-light-chain kinase [EC:2.7.11.18] | | | | | Down | 0.33 | -1.61 | 0.0038 |
|  |  | ko:K03903 | comp63621_c1_seq6 | | FGA; fibrinogen alpha chain | | | | | Up | 0.13 | -2.90 | 0.0195 |
|  |  | ko:K03904 | comp54730_c0_seq1 | | FGB; fibrinogen beta chain | | | | | Up | 2.33 | 1.22 | 0.0197 |
|  |  | ko:K03905 | comp40601_c0_seq1 | | FGG; fibrinogen gamma chain | | | | | Up | 3.94 | 1.98 | 0.0002 |
|  |  | ko:K04264 | comp66619_c0_seq9 | | TBXA2R; thromboxane A2 receptor | | | | | Down | 0.27 | -1.87 | 0.0063 |
|  |  | ko:K04270 | comp71548_c0_seq3 | | P2RY1; purinergic receptor P2Y, G protein-coupled, 1 | | | | | Down | 0.28 | -1.82 | 0.0014 |
|  |  | ko:K04298 | comp68923_c0_seq1 | | P2RY12; purinergic receptor P2Y, G protein-coupled, 12 | | | | | Down | 0.17 | -2.60 | 0.0004 |
|  |  | ko:K04350 | comp53987_c0_seq1 | | RASGRP1; RAS guanyl-releasing protein 1 | | | | | Down | 0.37 | -1.43 | 0.0269 |
|  |  | ko:K04958 | comp70776_c0_seq5 | | ITPR1; inositol 1,4,5-triphosphate receptor type 1 | | | | | Down | 0.27 | -1.89 | 0.0027 |
|  |  | ko:K06236 | comp48888_c0_seq2 | | COL1AS; collagen, type I/II/III/V/XI/XXIV/XXVII, alpha | | | | | Down | 0.27 | -1.87 | 0.0007 |
|  |  | ko:K06236 | comp57365_c0_seq1 | | COL1AS; collagen, type I/II/III/V/XI/XXIV/XXVII, alpha | | | | | Down | 0.23 | -2.12 | 0.0001 |
|  |  | ko:K06236 | comp69992_c3_seq3 | | COL1AS; collagen, type I/II/III/V/XI/XXIV/XXVII, alpha | | | | | Down | 0.36 | -1.46 | 0.0107 |
|  |  | ko:K06236 | comp70214_c0_seq2 | | COL1AS; collagen, type I/II/III/V/XI/XXIV/XXVII, alpha | | | | | Down | 0.23 | -2.10 | 0.0002 |
|  |  | ko:K06236 | comp70214_c0_seq3 | | COL1AS; collagen, type I/II/III/V/XI/XXIV/XXVII, alpha | | | | | Down | 0.24 | -2.09 | 0.0002 |
|  |  | ko:K06236 | comp71402_c0_seq5 | | COL1AS; collagen, type I/II/III/V/XI/XXIV/XXVII, alpha | | | | | Down | 0.29 | -1.80 | 0.0088 |
|  |  | ko:K06271 | comp68382_c1_seq1 | | TLN; talin | | | | | Down | 0.19 | -2.39 | 0.0003 |
|  |  | ko:K06271 | comp68382_c1_seq2 | | TLN; talin | | | | | Down | 0.19 | -2.38 | 0.0003 |
|  |  | ko:K07532 | comp71523_c0_seq1 | | ARHGEF12, LARG; Rho guanine nucleotide exchange factor 12 | | | | | Down | 0.30 | -1.75 | 0.0053 |
|  |  | ko:K08041 | comp25107_c0_seq1 | | ADCY1; adenylate cyclase 1 [EC:4.6.1.1] | | | | | Up | 170.33 | 7.41 | 0.0000 |
|  |  | ko:K08049 | comp70849_c0_seq2 | | ADCY9; adenylate cyclase 9 [EC:4.6.1.1] | | | | | Down | 0.36 | -1.48 | 0.0161 |
|  |  | ko:K12757 | comp46217_c0_seq1 | | MYL12; myosin regulatory light chain 12 | | | | | Up | Inf | Inf | 0.0265 |
|  |  | ko:K16342 | comp65623_c0_seq13 | | PLA2G4, CPLA2; cytosolic phospholipase A2 [EC:3.1.1.4] | | | | | Down | 0.25 | -2.01 | 0.0031 |
|  |  | ko:K16342 | comp67569_c2_seq20 | | PLA2G4, CPLA2; cytosolic phospholipase A2 [EC:3.1.1.4] | | | | | Down | 0.08 | -3.60 | 0.0000 |
| **ko04620** | **Toll-like receptor signaling pathway** | | | |  | | | | |  |  |  |  |
|  |  | ko:K01371 | comp62429_c0_seq1 | | CTSK; cathepsin K [EC:3.4.22.38] | | | | | Down | 0.44 | -1.18 | 0.0271 |
|  |  | ko:K03160 | comp70901_c3_seq1 | | TNFRSF5, CD40; tumor necrosis factor receptor superfamily member 5 | | | | | Down | 0.43 | -1.21 | 0.0301 |
|  |  | ko:K03160 | comp70901_c3_seq2 | | TNFRSF5, CD40; tumor necrosis factor receptor superfamily member 5 | | | | | Down | 0.39 | -1.36 | 0.0141 |
|  |  | ko:K04379 | comp66390_c3_seq1 | | FOS; proto-oncogene protein c-fos | | | | | Up | 0.22 | -2.16 | 0.0034 |
|  |  | ko:K04433 | comp40671_c0_seq1 | | MAP2K6, MKK6; mitogen-activated protein kinase kinase 6 [EC:2.7.12.2] | | | | | Down | 0.24 | -2.04 | 0.0190 |
|  |  | ko:K04519 | comp24971_c0_seq1 | | IL1B; interleukin 1 beta | | | | | Up | 5.12 | 2.36 | 0.0005 |
|  |  | ko:K05398 | comp69977_c1_seq4 | | TLR1; toll-like receptor 1 | | | | | Down | 0.22 | -2.19 | 0.0003 |
|  |  | ko:K05401 | comp70176_c0_seq2 | | TLR3; toll-like receptor 3 | | | | | Down | 0.34 | -1.58 | 0.0080 |
|  |  | ko:K05403 | comp67143_c0_seq1 | | TIRAP; toll-interleukin 1 receptor (TIR) domain-containing adaptor protein | | | | | Down | 0.46 | -1.11 | 0.0437 |
|  |  | ko:K05416 | comp43714_c0_seq1 | | CXCL9; C-X-C motif chemokine 9 | | | | | Down | 0.31 | -1.71 | 0.0017 |
|  |  | ko:K05416 | comp51337_c0_seq1 | | CXCL9; C-X-C motif chemokine 9 | | | | | Down | 0.37 | -1.42 | 0.0126 |
|  |  | ko:K05425 | comp16585_c0_seq1 | | IL12B; interleukin 12B | | | | | Down | 0.00 | #NAME? | 0.0013 |
|  |  | ko:K09447 | comp65743_c0_seq1 | | IRF7; interferon regulatory factor 7 | | | | | Down | 0.42 | -1.26 | 0.0356 |
|  |  | ko:K10030 | comp63378_c0_seq16 | | IL8, CXCL8; interleukin 8 | | | | | Down | 0.06 | -4.02 | 0.0010 |
|  |  | ko:K10159 | comp64223_c0_seq1 | | TLR2; toll-like receptor 2 | | | | | Down | 0.42 | -1.27 | 0.0336 |
|  |  | ko:K10161 | comp69734_c0_seq1 | | TLR9; toll-like receptor 9 | | | | | Down | 0.31 | -1.70 | 0.0301 |
|  |  | ko:K10168 | comp68815_c0_seq1 | | TLR5; toll-like receptor 5 | | | | | Up | 0.27 | -1.88 | 0.0067 |
|  |  | ko:K10170 | comp68922_c0_seq1 | | TLR8; toll-like receptor 8 | | | | | Down | 0.27 | -1.90 | 0.0031 |
|  |  | ko:K12671 | comp50472_c0_seq1 | | CXCL10, IP10; C-X-C motif chemokine 10 | | | | | Down | 0.20 | -2.33 | 0.0001 |
|  |  | ko:K12964 | comp56748_c0_seq1 | | CCL4; C-C motif chemokine 4 | | | | | Down | 0.20 | -2.32 | 0.0001 |
|  |  | ko:K12964 | comp60517_c1_seq2 | | CCL4; C-C motif chemokine 4 | | | | | Down | 0.26 | -1.95 | 0.0007 |
| **ko04621** | **NOD-like receptor signaling pathway** | | | | | | | | |  |  |  |  |
|  |  | ko:K04519 | comp24971_c0_seq1 | | IL1B; interleukin 1 beta | | | | | Up | 5.12 | 2.36 | 0.0005 |
|  |  | ko:K05482 | comp70179_c0_seq12 | | IL18, IL1F4; interleukin 18 | | | | | Down | 0.30 | -1.74 | 0.0065 |
|  |  | ko:K09487 | comp58660_c1_seq1 | | HSP90B, TRA1; heat shock protein 90kDa beta | | | | | Up | 0.41 | -1.30 | 0.0381 |
|  |  | ko:K09487 | comp54887_c0_seq1 | | HSP90B, TRA1; heat shock protein 90kDa beta | | | | | Up | Inf | Inf | 0.0000 |
|  |  | ko:K10030 | comp63378_c0_seq16 | | IL8, CXCL8; interleukin 8 | | | | | Down | 0.06 | -4.02 | 0.0010 |
| **ko04622** | **RIG-I-like receptor signaling pathway** | | | |  | | | | |  |  |  |  |
|  |  | ko:K05425 | comp16585_c0_seq1 | | IL12B; interleukin 12B | | | | | Down | 0.00 | #NAME? | 0.0013 |
|  |  | ko:K09447 | comp65743_c0_seq1 | | IRF7; interferon regulatory factor 7 | | | | | Down | 0.42 | -1.26 | 0.0356 |
|  |  | ko:K10030 | comp63378_c0_seq16 | | IL8, CXCL8; interleukin 8 | | | | | Down | 0.06 | -4.02 | 0.0010 |
|  |  | ko:K11594 | comp59178_c0_seq1 | | DDX3X, bel; ATP-dependent RNA helicase DDX3X [EC:3.6.4.13] | | | | | Up | 2.16 | 1.11 | 0.0378 |
|  |  | ko:K11594 | comp59178_c0_seq2 | | DDX3X, bel; ATP-dependent RNA helicase DDX3X [EC:3.6.4.13] | | | | | Up | 2.15 | 1.11 | 0.0381 |
|  |  | ko:K11594 | comp59178_c0_seq6 | | DDX3X, bel; ATP-dependent RNA helicase DDX3X [EC:3.6.4.13] | | | | | Up | 2.25 | 1.17 | 0.0288 |
|  |  | ko:K11594 | comp59178_c0_seq12 | | DDX3X, bel; ATP-dependent RNA helicase DDX3X [EC:3.6.4.13] | | | | | Up | 2.34 | 1.23 | 0.0219 |
|  |  | ko:K12671 | comp50472_c0_seq1 | | CXCL10, IP10; C-X-C motif chemokine 10 | | | | | Down | 0.20 | -2.33 | 0.0001 |
| **ko04623** | **Cytosolic DNA-sensing pathway** | | | | | | | | |  |  |  |  |
|  |  | ko:K03020 | comp64880_c0_seq1 | | RPC19, POLR1D; DNA-directed RNA polymerases I and III subunit RPAC2 | | | | | Up | 2.53 | 1.34 | 0.0228 |
|  |  | ko:K03026 | comp67161_c0_seq2 | | RPC53, POLR3D; DNA-directed RNA polymerase III subunit RPC4 | | | | | Up | 3.16 | 1.66 | 0.0081 |
|  |  | ko:K04519 | comp24971_c0_seq1 | | IL1B; interleukin 1 beta | | | | | Up | 3.16 | 1.66 | 0.0081 |
|  |  | ko:K05482 | comp70179_c0_seq12 | | IL18, IL1F4; interleukin 18 | | | | | Down | 5.12 | 2.36 | 0.0005 |
|  |  | ko:K09447 | comp65743_c0_seq1 | | IRF7; interferon regulatory factor 7 | | | | | Down | 0.42 | -1.26 | 0.0356 |
|  |  | ko:K12671 | comp50472_c0_seq1 | | CXCL10, IP10; C-X-C motif chemokine 10 | | | | | Down | 0.20 | -2.33 | 0.0001 |
|  |  | ko:K12964 | comp56748_c0_seq1 | | CCL4; C-C motif chemokine 4 | | | | | Down | 0.20 | -2.32 | 0.0001 |
|  |  | ko:K12964 | comp60517_c1_seq2 | | CCL4; C-C motif chemokine 4 | | | | | Down | 0.26 | -1.95 | 0.0007 |
| **ko04650** | **Natural killer cell mediated cytotoxicity** | | |  | |  |  |  |  |  |  |  |  |
|  |  | ko:K01353 | comp69662_c0_seq5 | | GZMB; granzyme B [EC:3.4.21.79] | | | | | Down | 0.20 | -2.32 | 0.0003 |
|  |  | ko:K01353 | comp70724_c2_seq2 | | GZMB; granzyme B [EC:3.4.21.79] | | | | | Down | 0.43 | -1.23 | 0.0401 |
|  |  | ko:K02187 | comp64282_c0_seq1 | | CASP3; caspase 3 [EC:3.4.22.56] | | | | | Up | 2.09 | 1.06 | 0.0487 |
|  |  | ko:K04721 | comp43070_c0_seq1 | | TNFSF10, TRAIL; tumor necrosis factor ligand superfamily member 10 | | | | | Down | 0.15 | -2.70 | 0.0227 |
|  |  | ko:K04721 | comp59230_c0_seq1 | | TNFSF10, TRAIL; tumor necrosis factor ligand superfamily member 10 | | | | | Down | 0.27 | -1.88 | 0.0157 |
|  |  | ko:K05730 | comp67167_c1_seq6 | | VAV; guanine nucleotide exchange factor VAV | | | | | Down | 0.21 | -2.27 | 0.0360 |
|  |  | ko:K05856 | comp54076_c0_seq1 | | LCK; lymphocyte cell-specific protein tyrosine kinase [EC:2.7.10.2] | | | | | Down | 0.32 | -1.66 | 0.0032 |
|  |  | ko:K06453 | comp17076_c0_seq1 | | CD3Z; CD3Z antigen, zeta polypeptide | | | | | Down | 0.38 | -1.39 | 0.0179 |
|  |  | ko:K06751 | comp71510_c1_seq1 | | MHC1; major histocompatibility complex, class I | | | | | Down | 0.43 | -1.23 | 0.0204 |
|  |  | ko:K07818 | comp60306_c1_seq26 | | PRF1; perforin 1 | | | | | Down | 0.39 | -1.35 | 0.0199 |
|  |  | ko:K07818 | comp65148_c0_seq2 | | PRF1; perforin 1 | | | | | Down | 0.16 | -2.62 | 0.0047 |
|  |  | ko:K07818 | comp70732_c0_seq1 | | PRF1; perforin 1 | | | | | Down | 0.27 | -1.90 | 0.0009 |
|  |  | ko:K07989 | comp31775_c0_seq1 | | EAT2; EWS/FLI1 activated transcript 2 | | | | | Down | 0.23 | -2.12 | 0.0008 |
|  |  | ko:K07990 | comp51398_c0_seq1 | | SH2D1A, SAP; SH2 domain protein 1A | | | | | Down | 0.24 | -2.08 | 0.0035 |
|  |  | ko:K07990 | comp70840_c0_seq1 | | SH2D1A, SAP; SH2 domain protein 1A | | | | | Down | 0.38 | -1.39 | 0.0130 |
|  |  | ko:K17332 | comp68768_c1_seq4 | | NFATC2, NFAT1, NFATP; nuclear factor of activated T-cells, cytoplasmic 2 | | | | | Down | 0.43 | -1.23 | 0.0402 |
|  |  | ko:K17447 | comp69980_c0_seq5 | | SHC2; SHC- transforming protein 2 | | | | | Up | 5.56 | 2.47 | 0.0000 |
|  |  | ko:K17449 | comp61832_c1_seq2 | | SHC4; SHC- transforming protein 4 | | | | | Up | 3.23 | 1.69 | 0.0345 |
| **ko04612** | **Antigen processing and presentation** | | | | | | | | |  |  |  |  |
|  |  | ko:K01365 | comp51540_c0_seq1 | | CTSL; cathepsin L [EC:3.4.22.15] | | | | | Up | 3.65 | 1.87 | 0.0005 |
|  |  | ko:K03283 | comp57103_c2_seq3 | | HSPA1_8; heat shock 70kDa protein 1/8 | | | | | Up | 4.80 | 2.26 | 0.0000 |
|  |  | ko:K03283 | comp57103_c2_seq7 | | HSPA1_8; heat shock 70kDa protein 1/8 | | | | | Up | 2.14 | 1.10 | 0.0362 |
|  |  | ko:K05653 | comp64421_c0_seq1 | | ABCB2, TAP1; ATP-binding cassette, subfamily B (MDR/TAP), member 2 | | | | | Down | 0.39 | -1.37 | 0.0141 |
|  |  | ko:K06458 | comp68785_c0_seq8 | | CD8A; CD8A antigen, alpha polypeptide | | | | | Down | 0.25 | -2.01 | 0.0009 |
|  |  | ko:K06505 | comp66019_c0_seq2 | | CD74, DHLAG; CD74 antigen | | | | | Down | 0.37 | -1.44 | 0.0062 |
|  |  | ko:K06697 | comp71207_c0_seq1 | | PSME2; proteasome activator subunit 2 (PA28 beta) | | | | | Up | 2.79 | 1.48 | 0.0124 |
|  |  | ko:K06698 | comp37484_c0_seq1 | | PSME3; proteasome activator subunit 3 (PA28 gamma) | | | | | Up | Inf | Inf | 0.0476 |
|  |  | ko:K06751 | comp71510_c1_seq1 | | MHC1; major histocompatibility complex, class I | | | | | Down | 0.43 | -1.23 | 0.0204 |
|  |  | ko:K06752 | comp68014_c0_seq2 | | MHC2; major histocompatibility complex, class II | | | | | Down | 0.21 | -2.25 | 0.0067 |
|  |  | ko:K06752 | comp69292_c0_seq2 | | MHC2; major histocompatibility complex, class II | | | | | Down | 0.36 | -1.45 | 0.0059 |
|  |  | ko:K06752 | comp71006_c1_seq1 | | MHC2; major histocompatibility complex, class II | | | | | Down | 0.38 | -1.41 | 0.0078 |
|  |  | ko:K06752 | comp71006_c1_seq5 | | MHC2; major histocompatibility complex, class II | | | | | Down | 0.38 | -1.40 | 0.0082 |
|  |  | ko:K06752 | comp71006_c1_seq6 | | MHC2; major histocompatibility complex, class II | | | | | Down | 0.38 | -1.40 | 0.0083 |
|  |  | ko:K08054 | comp70211_c0_seq1 | | CANX; calnexin | | | | | Up | 0.37 | -1.44 | 0.0122 |
|  |  | ko:K08055 | comp51071_c0_seq1 | | B2M; beta-2-microglobulin | | | | | Down | 0.48 | -1.07 | 0.0404 |
|  |  | ko:K08055 | comp58668_c1_seq1 | | B2M; beta-2-microglobulin | | | | | Down | 0.44 | -1.20 | 0.0230 |
|  |  | ko:K08056 | comp65883_c0_seq1 | | PDIA3, GRP58; protein disulfide isomerase family A, member 3 [EC:5.3.4.1] | | | | | Up | 2.23 | 1.16 | 0.0274 |
|  |  | ko:K08057 | comp54795_c1_seq1 | | CALR; calreticulin | | | | | Up | 2.18 | 1.12 | 0.0324 |
|  |  | ko:K08058 | comp66709_c0_seq2 | | TAPBP; TAP binding protein (tapasin) | | | | | Down | 0.40 | -1.31 | 0.0137 |
|  |  | ko:K08059 | comp56841_c0_seq1 | | IFI30, GILT; interferon, gamma-inducible protein 30 | | | | | Down | 0.34 | -1.56 | 0.0033 |
|  |  | ko:K08060 | comp64332_c0_seq1 | | CIITA; class II, major histocompatibility complex, transactivator | | | | | Down | 0.26 | -1.97 | 0.0011 |
|  |  | ko:K09489 | comp64204_c0_seq1 | | HSPA4; heat shock 70kDa protein 4 | | | | | Up | 0.34 | -1.54 | 0.0091 |
| **ko04660** | **T cell receptor signaling pathway** | | | | | | | | |  |  |  |  |
|  |  | ko:K04350 | comp53987_c0_seq1 | | RASGRP1; RAS guanyl-releasing protein 1 | | | | | Down | 0.37 | -1.43 | 0.0269 |
|  |  | ko:K04379 | comp66390_c3_seq1 | | FOS; proto-oncogene protein c-fos | | | | | Up | 2.30 | 1.20 | 0.0269 |
|  |  | ko:K05730 | comp67167_c1_seq6 | | VAV; guanine nucleotide exchange factor VAV | | | | | Down | 0.21 | -2.27 | 0.0360 |
|  |  | ko:K05856 | comp54076_c0_seq1 | | LCK; lymphocyte cell-specific protein tyrosine kinase [EC:2.7.10.2] | | | | | Down | 0.32 | -1.66 | 0.0032 |
|  |  | ko:K06451 | comp68592_c0_seq1 | | CD3E; T-cell surface glycoprotein CD3 epsilon chain | | | | | Down | 0.25 | -1.99 | 0.0017 |
|  |  | ko:K06451 | comp68592_c0_seq3 | | CD3E; T-cell surface glycoprotein CD3 epsilon chain | | | | | Down | 0.33 | -1.61 | 0.0110 |
|  |  | ko:K06453 | comp17076_c0_seq1 | | CD3Z; CD3Z antigen, zeta polypeptide | | | | | Down | 0.38 | -1.39 | 0.0179 |
|  |  | ko:K06458 | comp68785_c0_seq8 | | CD8A; CD8A antigen, alpha polypeptide | | | | | Down | 0.25 | -2.01 | 0.0009 |
|  |  | ko:K07363 | comp70960_c0_seq6 | | ITK; IL2-inducible T-cell kinase [EC:2.7.10.2] | | | | | Down | 0.37 | -1.43 | 0.0088 |
|  |  | ko:K17332 | comp68768_c1_seq4 | | NFATC2, NFAT1, NFATP; nuclear factor of activated T-cells, cytoplasmic 2 | | | | | Down | 0.43 | -1.23 | 0.0402 |
|  |  | ko:K18052 | comp61291_c0_seq1 | | PRKCQ; novel protein kinase C theta type [EC:2.7.11.13] | | | | | Down | 0.41 | -1.28 | 0.0380 |
| **ko04662** | **B cell receptor signaling pathway** | | | | | | | | |  |  |  |  |
|  |  | ko:K04379 | comp66390_c3_seq1 | | FOS; proto-oncogene protein c-fos | | | | | Up | 2.30 | 1.20 | 0.0269 |
|  |  | ko:K05730 | comp67167_c1_seq6 | | VAV; guanine nucleotide exchange factor VAV | | | | | Down | 0.21 | -2.27 | 0.0360 |
|  |  | ko:K06467 | comp70355_c2_seq3 | | CD22, SIGLEC2; CD22 antigen | | | | | Down | 0.28 | -1.83 | 0.0011 |
|  |  | ko:K06507 | comp53870_c0_seq1 | | CD79B, IGB; CD79B antigen | | | | | Down | 0.33 | -1.60 | 0.0055 |
|  |  | ko:K17332 | comp68768_c1_seq4 | | NFATC2, NFAT1, NFATP; nuclear factor of activated T-cells, cytoplasmic 2 | | | | | Down | 0.43 | -1.23 | 0.0402 |
| **ko04664** | **Fc epsilon RI signaling pathway** | | | | | | | | |  |  |  |  |
|  |  | ko:K04433 | comp40671_c0_seq1 | | MAP2K6, MKK6; mitogen-activated protein kinase kinase 6 [EC:2.7.12.2] | | | | | Down | 0.24 | -2.04 | 0.0190 |
|  |  | ko:K05730 | comp67167_c1_seq6 | | VAV; guanine nucleotide exchange factor VAV | | | | | Down | 0.21 | -2.27 | 0.0360 |
|  |  | ko:K16342 | comp65623_c0_seq13 | | PLA2G4, CPLA2; cytosolic phospholipase A2 [EC:3.1.1.4] | | | | | Down | 0.25 | -2.01 | 0.0031 |
|  |  | ko:K16342 | comp67569_c2_seq20 | | PLA2G4, CPLA2; cytosolic phospholipase A2 [EC:3.1.1.4] | | | | | Down | 0.08 | -3.60 | 0.0000 |
| **ko04666** | **Fc gamma R-mediated phagocytosis** | | | | | | | | |  |  |  |  |
|  |  | ko:K01080 | comp59984_c0_seq2 | | PPAP2; phosphatidate phosphatase [EC:3.1.3.4] | | | | | Down | 0.40 | -1.32 | 0.0150 |
|  |  | ko:K05730 | comp67167_c1_seq6 | | VAV; guanine nucleotide exchange factor VAV | | | | | Down | 0.21 | -2.27 | 0.0360 |
|  |  | ko:K05768 | comp61214_c0_seq12 | | GSN; gelsolin | | | | | Down | 0.28 | -1.82 | 0.0042 |
|  |  | ko:K12488 | comp71477_c2_seq1 | | ASAP; Arf-GAP with SH3 domain, ANK repeat and PH domain-containing protein | | | | | Down | 0.25 | -2.02 | 0.0006 |
|  |  | ko:K12559 | comp70974_c1_seq2 | | MYO10; myosin X | | | | | Down | 0.33 | -1.59 | 0.0075 |
|  |  | ko:K12561 | comp63633_c0_seq1 | | MARCKS; myristoylated alanine-rich C-kinase substrate | | | | | Down | 0.33 | -1.59 | 0.0075 |
|  |  | ko:K12562 | comp51686_c0_seq3 | | AMPH; amphiphysin | | | | | Down | 0.28 | -1.86 | 0.0218 |
|  |  | ko:K13536 | comp55572_c0_seq1 | | MARCKSL1, MRP; MARCKS-related protein | | | | | Down | 0.38 | -1.41 | 0.0095 |
|  |  | ko:K13536 | comp68629_c0_seq1 | | MARCKSL1, MRP; MARCKS-related protein | | | | | Down | 0.27 | -1.90 | 0.0025 |
| **ko04670** | **Leukocyte transendothelial migration** | | | |  | | | | |  |  |  |  |
|  |  | ko:K01398 | comp66876_c0_seq1 | | MMP2; matrix metalloproteinase-2 (gelatinase A) [EC:3.4.24.24] | | | | | Down | 0.25 | -2.02 | 0.0005 |
|  |  | ko:K01403 | comp54851_c0_seq1 | | MMP9; matrix metalloproteinase-9 (gelatinase B) [EC:3.4.24.35] | | | | | Up | 7.45 | 2.90 | 0.0000 |
|  |  | ko:K05730 | comp67167_c1_seq6 | | VAV; guanine nucleotide exchange factor VAV | | | | | Down | 0.21 | -2.27 | 0.0360 |
|  |  | ko:K06087 | comp54074_c0_seq1 | | CLDN; claudin | | | | | Down | 0.22 | -2.18 | 0.0451 |
|  |  | ko:K06087 | comp57663_c0_seq2 | | CLDN; claudin | | | | | Down | 0.34 | -1.56 | 0.0158 |
|  |  | ko:K06087 | comp34636_c0_seq1 | | CLDN; claudin | | | | | Up | 39.77 | 5.31 | 0.0147 |
|  |  | ko:K06087 | comp56654_c0_seq1 | | CLDN; claudin | | | | | Up | 2.15 | 1.10 | 0.0424 |
|  |  | ko:K06087 | comp62297_c0_seq1 | | CLDN; claudin | | | | | Up | 2.84 | 1.51 | 0.0069 |
|  |  | ko:K06089 | comp65654_c0_seq2 | | F11R, JAM1; junctional adhesion molecule 1 | | | | | Up | 2.76 | 1.46 | 0.0102 |
|  |  | ko:K06514 | comp30604_c0_seq1 | | THY1, CD90; Thy-1 cell surface antigen | | | | | Down | 0.14 | -2.79 | 0.0002 |
|  |  | ko:K06527 | comp54821_c0_seq4 | | VCAM1; vascular cell adhesion molecule 1 | | | | | Down | 0.30 | -1.74 | 0.0036 |
|  |  | ko:K06527 | comp54821_c0_seq5 | | VCAM1; vascular cell adhesion molecule 1 | | | | | Down | 0.22 | -2.16 | 0.0004 |
|  |  | ko:K07363 | comp70960_c0_seq6 | | ITK; IL2-inducible T-cell kinase [EC:2.7.10.2] | | | | | Down | 0.37 | -1.43 | 0.0088 |
|  |  | ko:K07873 | comp70779_c0_seq3 | | RHOH, TTF; Ras homolog gene family, member H | | | | | Down | 0.43 | -1.22 | 0.0440 |
|  |  | ko:K10031 | comp62570_c2_seq1 | | CXCL12; C-X-C motif chemokine 12 | | | | | Down | 0.07 | -3.86 | 0.0000 |
|  |  | ko:K12755 | comp67921_c0_seq1 | | MYL9; myosin regulatory light chain 9 | | | | | Down | 0.34 | -1.57 | 0.0035 |
|  |  | ko:K12757 | comp46217_c0_seq1 | | MYL12; myosin regulatory light chain 12 | | | | | Up | Inf | Inf | 0.0265 |
| **ko04672** | **Intestinal immune network for IgA production** | | | |  | | | | |  |  |  |  |
|  |  | ko:K03160 | comp70901_c3_seq1 | | TNFRSF5, CD40; tumor necrosis factor receptor superfamily member 5 | | | | | Down | 0.43 | -1.21 | 0.0301 |
|  |  | ko:K03160 | comp70901_c3_seq2 | | TNFRSF5, CD40; tumor necrosis factor receptor superfamily member 5 | | | | | Down | 0.39 | -1.36 | 0.0141 |
|  |  | ko:K05150 | comp65682_c0_seq1 | | TNFRSF13B, TACI; tumor necrosis factor receptor superfamily member 13B | | | | | Down | 0.37 | -1.45 | 0.0188 |
|  |  | ko:K06752 | comp68014_c0_seq2 | | MHC2; major histocompatibility complex, class II | | | | | Down | 0.21 | -2.25 | 0.0067 |
|  |  | ko:K06752 | comp69292_c0_seq2 | | MHC2; major histocompatibility complex, class II | | | | | Down | 0.36 | -1.45 | 0.0059 |
|  |  | ko:K06752 | comp71006_c1_seq1 | | MHC2; major histocompatibility complex, class II | | | | | Down | 0.38 | -1.41 | 0.0078 |
|  |  | ko:K06752 | comp71006_c1_seq5 | | MHC2; major histocompatibility complex, class II | | | | | Down | 0.38 | -1.40 | 0.0082 |
|  |  | ko:K06752 | comp71006_c1_seq6 | | MHC2; major histocompatibility complex, class II | | | | | Down | 0.38 | -1.40 | 0.0083 |
|  |  | ko:K10031 | comp62570_c2_seq1 | | CXCL12; C-X-C motif chemokine 12 | | | | | Down | 0.07 | -3.86 | 0.0000 |
|  |  | ko:K13073 | comp64030_c1_seq2 | | PIGR; polymeric immunoglobulin receptor | | | | | Down | 0.38 | -1.39 | 0.0196 |
|  |  | ko:K13073 | comp64030_c1_seq5 | | PIGR; polymeric immunoglobulin receptor | | | | | Down | 0.36 | -1.48 | 0.0124 |
| **ko04062** | **Chemokine signaling pathway** | | | | | | | | |  |  |  |  |
|  |  | ko:K04180 | comp63335_c0_seq1 | | CCR5; C-C chemokine receptor type 5 | | | | | Down | 0.25 | -1.98 | 0.0007 |
|  |  | ko:K04182 | comp28871_c0_seq2 | | CCR7; C-C chemokine receptor type 7 | | | | | Down | 0.36 | -1.48 | 0.0142 |
|  |  | ko:K04182 | comp28871_c0_seq3 | | CCR7; C-C chemokine receptor type 7 | | | | | Down | 0.38 | -1.38 | 0.0213 |
|  |  | ko:K04182 | comp52400_c0_seq2 | | CCR7; C-C chemokine receptor type 7 | | | | | Down | 0.20 | -2.31 | 0.0082 |
|  |  | ko:K04188 | comp58887_c0_seq1 | | CXCR3, GPR9; C-X-C chemokine receptor type 3 | | | | | Down | 0.44 | -1.19 | 0.0391 |
|  |  | ko:K04188 | comp62959_c0_seq1 | | CXCR3, GPR9; C-X-C chemokine receptor type 3 | | | | | Down | 0.38 | -1.40 | 0.0100 |
|  |  | ko:K04190 | comp66135_c0_seq7 | | CXCR5, BLR1; C-X-C chemokine receptor type 5 | | | | | Down | 0.39 | -1.36 | 0.0248 |
|  |  | ko:K04347 | comp65261_c6_seq1 | | GNG12; guanine nucleotide-binding protein G(I)/G(S)/G(O) subunit gamma-12 | | | | | Up | 5.00 | 2.32 | 0.0000 |
|  |  | ko:K05050 | comp40762_c0_seq1 | | IL8RB, CXCR2; interleukin 8 receptor beta | | | | | Down | 0.28 | -1.86 | 0.0170 |
|  |  | ko:K05050 | comp71229_c1_seq1 | | IL8RB, CXCR2; interleukin 8 receptor beta | | | | | Down | 0.11 | -3.12 | 0.0006 |
|  |  | ko:K05416 | comp43714_c0_seq1 | | CXCL9; C-X-C motif chemokine 9 | | | | | Down | 0.31 | -1.71 | 0.0017 |
|  |  | ko:K05416 | comp51337_c0_seq1 | | CXCL9; C-X-C motif chemokine 9 | | | | | Down | 0.37 | -1.42 | 0.0126 |
|  |  | ko:K05506 | comp60004_c0_seq1 | | CXCL5_6, SCYB5_6; C-X-C motif chemokine 5/6 | | | | | Down | 0.44 | -1.19 | 0.0393 |
|  |  | ko:K05512 | comp59357_c0_seq1 | | CCL19, ELC; C-C motif chemokine 19 | | | | | Down | 0.14 | -2.84 | 0.0034 |
|  |  | ko:K05730 | comp67167_c1_seq6 | | VAV; guanine nucleotide exchange factor VAV | | | | | Down | 0.21 | -2.27 | 0.0360 |
|  |  | ko:K07363 | comp70960_c0_seq6 | | ITK; IL2-inducible T-cell kinase [EC:2.7.10.2] | | | | | Down | 0.37 | -1.43 | 0.0088 |
|  |  | ko:K08041 | comp25107_c0_seq1 | | ADCY1; adenylate cyclase 1 [EC:4.6.1.1] | | | | | Up | 170.33 | 7.41 | 0.0000 |
|  |  | ko:K08049 | comp70849_c0_seq2 | | ADCY9; adenylate cyclase 9 [EC:4.6.1.1] | | | | | Down | 0.36 | -1.48 | 0.0161 |
|  |  | ko:K10030 | comp63378_c0_seq16 | | IL8, CXCL8; interleukin 8 | | | | | Down | 0.06 | -4.02 | 0.0010 |
|  |  | ko:K10031 | comp62570_c2_seq1 | | CXCL12; C-X-C motif chemokine 12 | | | | | Down | 0.07 | -3.86 | 0.0000 |
|  |  | ko:K12671 | comp50472_c0_seq1 | | CXCL10, IP10; C-X-C motif chemokine 10 | | | | | Down | 0.20 | -2.33 | 0.0001 |
|  |  | ko:K12964 | comp56748_c0_seq1 | | CCL4; C-C motif chemokine 4 | | | | | Down | 0.20 | -2.32 | 0.0001 |
|  |  | ko:K12964 | comp60517_c1_seq2 | | CCL4; C-C motif chemokine 4 | | | | | Down | 0.26 | -1.95 | 0.0007 |
|  |  | ko:K14625 | comp50234_c0_seq1 | | CCL20; C-C motif chemokine 20 | | | | | Up | 4.60 | 2.20 | 0.0007 |
|  |  | ko:K17447 | comp69980_c0_seq5 | | SHC2; SHC- transforming protein 2 | | | | | Up | 5.56 | 2.47 | 0.0000 |
|  |  | ko:K17449 | comp61832_c1_seq2 | | SHC4; SHC- transforming protein 4 | | | | | Up | 3.23 | 1.69 | 0.0345 |
|  |  |  |  | |  | | | | |  |  |  |  |
| **C vs A** |  |  |  | |  | | | | |  |  |  |  |
| **ko04640** | **Hematopoietic cell lineage** | | | | | | | | |  |  |  |  |
|  |  | ko:K05417 | comp59437_c0_seq4 | | IL11; interleukin 11 | | | | | Down | 0.33 | -1.58 | 0.0207 |
|  |  | ko:K05423 | comp64110_c1_seq2 | | CSF3, GCSF; granulocyte colony-stimulating factor | | | | | Down | 0.32 | -1.63 | 0.0109 |
|  |  | ko:K06449 | comp68860_c0_seq1 | | CD2; CD2 antigen | | | | | Down | 0.32 | -1.65 | 0.0130 |
|  |  | ko:K06460 | comp62472_c0_seq1 | | CD9, TSPAN29; CD9 antigen | | | | | Down | 0.26 | -1.96 | 0.0404 |
| **ko04610** | **Complement and coagulation cascades** | | | | | | | | |  |  |  |  |
|  |  | ko:K01333 | comp64792_c0_seq2 | | CFI; complement factor I [EC:3.4.21.45] | | | | | Up | 2.42 | 1.28 | 0.0165 |
|  |  | ko:K01343 | comp69115_c0_seq2 | | PLAT; tissue plasminogen activator [EC:3.4.21.68] | | | | | Down | 0.44 | -1.17 | 0.0464 |
|  |  | ko:K01343 | comp69115_c0_seq4 | | PLAT; tissue plasminogen activator [EC:3.4.21.68] | | | | | Down | 0.44 | -1.17 | 0.0450 |
|  |  | ko:K03907 | comp66488_c1_seq7 | | THBD, CD141; thrombomodulin | | | | | Down | 0.38 | -1.40 | 0.0191 |
|  |  | ko:K03907 | comp70211_c1_seq13 | | THBD, CD141; thrombomodulin | | | | | Down | 0.34 | -1.54 | 0.0079 |
|  |  | ko:K03991 | comp58993_c0_seq3 | | MBL; mannose-binding lectin | | | | | Down | 0.19 | -2.39 | 0.0001 |
|  |  | ko:K03996 | comp71185_c1_seq1 | | C7; complement component 7 | | | | | Up | 2.90 | 1.54 | 0.0041 |
|  |  | ko:K04000 | comp66790_c0_seq2 | | C9; complement component 9 | | | | | Up | 3.18 | 1.67 | 0.0019 |
| **ko04611** | **Platelet activation** | | | |  | | | | |  |  |  |  |
|  |  | ko:K00907 | comp29446_c0_seq1 | | MYLK; myosin-light-chain kinase [EC:2.7.11.18] | | | | | Down | 0.36 | -1.48 | 0.0082 |
|  |  | ko:K06236 | comp48888_c0_seq2 | | COL1AS; collagen, type I/II/III/V/XI/XXIV/XXVII, alpha | | | | | Down | 0.33 | -1.58 | 0.0040 |
|  |  | ko:K06236 | comp52119_c0_seq1 | | COL1AS; collagen, type I/II/III/V/XI/XXIV/XXVII, alpha | | | | | Down | 0.09 | -3.51 | 0.0004 |
|  |  | ko:K06236 | comp57365_c0_seq1 | | COL1AS; collagen, type I/II/III/V/XI/XXIV/XXVII, alpha | | | | | Down | 0.34 | -1.54 | 0.0047 |
|  |  | ko:K06236 | comp69992_c3_seq3 | | COL1AS; collagen, type I/II/III/V/XI/XXIV/XXVII, alpha | | | | | Down | 0.32 | -1.63 | 0.0048 |
|  |  | ko:K06236 | comp70214_c0_seq2 | | COL1AS; collagen, type I/II/III/V/XI/XXIV/XXVII, alpha | | | | | Down | 0.41 | -1.28 | 0.0208 |
|  |  | ko:K06236 | comp70214_c0_seq3 | | COL1AS; collagen, type I/II/III/V/XI/XXIV/XXVII, alpha | | | | | Down | 0.29 | -1.78 | 0.0016 |
|  |  | ko:K06236 | comp71402_c0_seq5 | | COL1AS; collagen, type I/II/III/V/XI/XXIV/XXVII, alpha | | | | | Down | 0.26 | -1.93 | 0.0057 |
|  |  | ko:K06271 | comp68382_c1_seq1 | | TLN; talin | | | | | Down | 0.19 | -2.41 | 0.0003 |
|  |  | ko:K06271 | comp68382_c1_seq2 | | TLN; talin | | | | | Down | 0.20 | -2.35 | 0.0005 |
|  |  | ko:K07532 | comp71523_c0_seq1 | | ARHGEF12, LARG; Rho guanine nucleotide exchange factor 12 | | | | | Down | 0.33 | -1.59 | 0.0113 |
|  |  | ko:K08041 | comp25107_c0_seq1 | | ADCY1; adenylate cyclase 1 [EC:4.6.1.1] | | | | | Up | 14.85 | 3.89 | 0.0044 |
|  |  | ko:K16342 | comp65623_c0_seq13 | | PLA2G4, CPLA2; cytosolic phospholipase A2 [EC:3.1.1.4] | | | | | Down | 0.26 | -1.95 | 0.0042 |
|  |  | ko:K16342 | comp67569_c2_seq20 | | PLA2G4, CPLA2; cytosolic phospholipase A2 [EC:3.1.1.4] | | | | | Down | 0.01 | -6.17 | 0.0000 |
| **ko04620** | **Toll-like receptor signaling pathway** | | | |  | | | | |  |  |  |  |
|  |  | ko:K05398 | comp6353_c0_seq1 | | TLR1; toll-like receptor 1 | | | | | Down | 0.15 | -2.73 | 0.0040 |
|  |  | ko:K05416 | comp51337_c0_seq1 | | CXCL9; C-X-C motif chemokine 9 | | | | | Down | 0.29 | -1.78 | 0.0023 |
|  |  | ko:K05425 | comp16585_c0_seq1 | | IL12B; interleukin 12B | | | | | Down | 0.03 | -4.87 | 0.0129 |
|  |  | ko:K10030 | comp51351_c0_seq5 | | IL8, CXCL8; interleukin 8 | | | | | Down | 0.20 | -2.30 | 0.0007 |
|  |  | ko:K10030 | comp56058_c0_seq1 | | IL8, CXCL8; interleukin 8 | | | | | Down | 0.28 | -1.85 | 0.0053 |
|  |  | ko:K10030 | comp63378_c0_seq16 | | IL8, CXCL8; interleukin 8 | | | | | Down | 0.07 | -3.89 | 0.0014 |
|  |  | ko:K12671 | comp50472_c0_seq1 | | CXCL10, IP10; C-X-C motif chemokine 10 | | | | | Down | 0.40 | -1.33 | 0.0228 |
| **ko04621** | **NOD-like receptor signaling pathway** | | | | | | | | |  |  |  |  |
|  |  | ko:K10030 | comp51351_c0_seq5 | | IL8, CXCL8; interleukin 8 | | | | | Down | 0.20 | -2.30 | 0.0007 |
|  |  | ko:K10030 | comp56058_c0_seq1 | | IL8, CXCL8; interleukin 8 | | | | | Down | 0.28 | -1.85 | 0.0053 |
|  |  | ko:K10030 | comp63378_c0_seq16 | | IL8, CXCL8; interleukin 8 | | | | | Down | 0.07 | -3.89 | 0.0014 |
| **ko04622** | **RIG-I-like receptor signaling pathway** | | | |  | | | | |  |  |  |  |
|  |  | ko:K05425 | comp16585_c0_seq1 | | IL12B; interleukin 12B | | | | | Down | 0.03 | -4.87 | 0.0129 |
|  |  | ko:K10030 | comp51351_c0_seq5 | | IL8, CXCL8; interleukin 8 | | | | | Down | 0.20 | -2.30 | 0.0007 |
|  |  | ko:K10030 | comp56058_c0_seq1 | | IL8, CXCL8; interleukin 8 | | | | | Down | 0.28 | -1.85 | 0.0053 |
|  |  | ko:K10030 | comp63378_c0_seq16 | | IL8, CXCL8; interleukin 8 | | | | | Down | 0.07 | -3.89 | 0.0014 |
|  |  | ko:K12671 | comp50472_c0_seq1 | | CXCL10, IP10; C-X-C motif chemokine 10 | | | | | Down | 0.40 | -1.33 | 0.0228 |
| **ko04623** | **Cytosolic DNA-sensing pathway** | | | | | | | | |  |  |  |  |
|  |  | ko:K12671 | comp50472_c0_seq1 | | CXCL10, IP10; C-X-C motif chemokine 10 | | | | | Down | 0.40 | -1.33 | 0.0228 |
| **ko04650** | **Natural killer cell mediated cytotoxicity** | | | |  | | | | |  |  |  |  |
|  |  | ko:K06453 | comp17076_c0_seq1 | | CD3Z; CD3Z antigen, zeta polypeptide | | | | | Up | 2.41 | 1.27 | 0.0301 |
| **ko04612** | **Antigen processing and presentation** | | | | | | | | |  |  |  |  |
|  |  | ko:K03283 | comp57103_c2_seq3 | | HSPA1_8; heat shock 70kDa protein 1/8 | | | | | Up | 5.08 | 2.34 | 0.0000 |
|  |  | ko:K03283 | comp57103_c2_seq7 | | HSPA1_8; heat shock 70kDa protein 1/8 | | | | | Up | 2.20 | 1.14 | 0.0324 |
|  |  | ko:K09489 | comp64204_c0_seq1 | | HSPA4; heat shock 70kDa protein 4 | | | | | Up | 3.39 | 1.76 | 0.0011 |
| **ko04660** | **T cell receptor signaling pathway** | | | | | | | | |  |  |  |  |
|  |  | ko:K06453 | comp17076_c0_seq1 | | CD3Z; CD3Z antigen, zeta polypeptide | | | | | Up | 2.41 | 1.27 | 0.0301 |
| **ko04662** | **B cell receptor signaling pathway** | | | | | | | | |  |  |  |  |
| **ko04664** | **Fc epsilon RI signaling pathway** | | | | | | | | |  |  |  |  |
|  |  | ko:K16342 | comp65623_c0_seq13 | | PLA2G4, CPLA2; cytosolic phospholipase A2 [EC:3.1.1.4] | | | | | Down | 0.26 | -1.95 | 0.0042 |
|  |  | ko:K16342 | comp67569_c2_seq20 | | PLA2G4, CPLA2; cytosolic phospholipase A2 [EC:3.1.1.4] | | | | | Down | 0.01 | -6.17 | 0.0000 |
| **ko04666** | **Fc gamma R-mediated phagocytosis** | | | | | | | | |  |  |  |  |
|  |  | ko:K12559 | comp70974_c1_seq2 | | MYO10; myosin X | | | | | Down | 0.41 | -1.28 | 0.0320 |
|  |  | ko:K13536 | comp55572_c0_seq1 | | MARCKSL1, MRP; MARCKS-related protein | | | | | Down | 0.46 | -1.11 | 0.0423 |
| **ko04670** | **Leukocyte transendothelial migration** | | | |  | | | | |  |  |  |  |
|  |  | ko:K01398 | comp66876_c0_seq1 | | MMP2; matrix metalloproteinase-2 (gelatinase A) [EC:3.4.24.24] | | | | | Down | 0.23 | -2.12 | 0.0003 |
|  |  | ko:K06514 | comp30604_c0_seq1 | | THY1, CD90; Thy-1 cell surface antigen | | | | | Down | 0.20 | -2.29 | 0.0019 |
|  |  | ko:K10031 | comp62570_c2_seq1 | | CXCL12; C-X-C motif chemokine 12 | | | | | Down | 0.34 | -1.57 | 0.0202 |
|  |  | ko:K12755 | comp67921_c0_seq1 | | MYL9; myosin regulatory light chain 9 | | | | | Down | 0.45 | -1.17 | 0.0301 |
|  |  | ko:K12758 | comp64656_c0_seq4 | | MYLPF; fast skeletal myosin light chain 2 | | | | | Up | Inf | Inf | 0.0092 |
| **ko04672** | **Intestinal immune network for IgA production** | | | |  | | | | |  |  |  |  |
|  |  | ko:K10031 | comp62570_c2_seq1 | | CXCL12; C-X-C motif chemokine 12 | | | | | Down | 0.34 | -1.57 | 0.0202 |
|  |  | ko:K10989 | comp50258_c0_seq1 | | AICDA, AID; activation-induced cytidine deaminase [EC:3.5.4.38] | | | | | Up | 8.13 | 3.02 | 0.0092 |
| **ko04062** | **Chemokine signaling pathway** | | | | | | | | |  |  |  |  |
|  |  | ko:K05050 | comp40762_c0_seq1 | | IL8RB, CXCR2; interleukin 8 receptor beta | | | | | Down | 0.19 | -2.40 | 0.0027 |
|  |  | ko:K05416 | comp51337_c0_seq1 | | CXCL9; C-X-C motif chemokine 9 | | | | | Down | 0.29 | -1.78 | 0.0023 |
|  |  | ko:K05512 | comp51567_c0_seq1 | | CCL19, ELC; C-C motif chemokine 19 | | | | | Down | 0.47 | -1.08 | 0.0436 |
|  |  | ko:K08041 | comp25107_c0_seq1 | | ADCY1; adenylate cyclase 1 [EC:4.6.1.1] | | | | | Up | 14.85 | 3.89 | 0.0044 |
|  |  | ko:K10030 | comp51351_c0_seq5 | | IL8, CXCL8; interleukin 8 | | | | | Down | 0.20 | -2.30 | 0.0007 |
|  |  | ko:K10030 | comp56058_c0_seq1 | | IL8, CXCL8; interleukin 8 | | | | | Down | 0.28 | -1.85 | 0.0053 |
|  |  | ko:K10030 | comp63378_c0_seq16 | | IL8, CXCL8; interleukin 8 | | | | | Down | 0.07 | -3.89 | 0.0014 |
|  |  | ko:K10031 | comp62570_c2_seq1 | | CXCL12; C-X-C motif chemokine 12 | | | | | Down | 0.34 | -1.57 | 0.0202 |
|  |  | ko:K12671 | comp50472_c0_seq1 | | CXCL10, IP10; C-X-C motif chemokine 10 | | | | | Down | 0.40 | -1.33 | 0.0228 |
|  |  |  |  | |  | | | | |  |  |  |  |
| **D vs A** |  |  |  | |  | | | | |  |  |  |  |
| **ko04640** | **Hematopoietic cell lineage** | | | | | | | | |  |  |  |  |
|  |  | ko:K00977 | comp60169_c1_seq1 | | E2.7.7.31, DNTT; DNA nucleotidylexotransferase [EC:2.7.7.31] | | | | | Up | 3.86 | 1.95 | 0.0392 |
|  |  | ko:K04008 | comp18297_c0_seq1 | | CD59; CD59 antigen | | | | | Up | 3.39 | 1.76 | 0.0242 |
|  |  | ko:K04008 | comp54684_c0_seq1 | | CD59; CD59 antigen | | | | | Up | 3.63 | 1.86 | 0.0006 |
|  |  | ko:K04008 | comp57062_c0_seq1 | | CD59; CD59 antigen | | | | | Up | 4.18 | 2.06 | 0.0002 |
|  |  | ko:K05072 | comp61812_c0_seq7 | | IL7R; interleukin 7 receptor | | | | | Down | 0.41 | -1.30 | 0.0336 |
|  |  | ko:K05092 | comp71006_c0_seq1 | | FLT3, FLK2; fms-related tyrosine kinase 3 [EC:2.7.10.1] | | | | | Down | 0.45 | -1.15 | 0.0311 |
|  |  | ko:K06261 | comp67565_c7_seq4 | | GP1BA; platelet glycoprotein Ib alpha chain | | | | | Up | 2.39 | 1.26 | 0.0184 |
|  |  | ko:K06454 | comp70556_c0_seq2 | | CD4; CD4 antigen | | | | | Down | 0.41 | -1.29 | 0.0301 |
|  |  | ko:K06458 | comp68785_c0_seq8 | | CD8A; CD8A antigen, alpha polypeptide | | | | | Down | 0.38 | -1.38 | 0.0198 |
|  |  | ko:K06467 | comp70355_c2_seq3 | | CD22, SIGLEC2; CD22 antigen | | | | | Down | 0.48 | -1.07 | 0.0497 |
|  |  | ko:K06498 | comp56824_c0_seq1 | | FCGR1A, CD64; high affinity immunoglobulin gamma Fc receptor I | | | | | Down | 0.48 | -1.06 | 0.0446 |
| **ko04610** | **Complement and coagulation cascades** | | | | | | | | |  |  |  |  |
|  |  | ko:K01300 | comp65823_c0_seq1 | | CPB2; carboxypeptidase B2 [EC:3.4.17.20] | | | | | Up | 3.62 | 1.85 | 0.0007 |
|  |  | ko:K01313 | comp32288_c0_seq1 | | F2; coagulation factor II (thrombin) [EC:3.4.21.5] | | | | | Up | 2.16 | 1.11 | 0.0342 |
|  |  | ko:K01315 | comp58611_c0_seq1 | | PLG; plasminogen [EC:3.4.21.7] | | | | | Up | 2.43 | 1.28 | 0.0148 |
|  |  | ko:K01320 | comp43168_c0_seq1 | | F7; coagulation factor VII [EC:3.4.21.21] | | | | | Up | 2.43 | 1.28 | 0.0174 |
|  |  | ko:K01320 | comp54518_c0_seq1 | | F7; coagulation factor VII [EC:3.4.21.21] | | | | | Up | 2.59 | 1.37 | 0.0113 |
|  |  | ko:K01320 | comp57677_c0_seq1 | | F7; coagulation factor VII [EC:3.4.21.21] | | | | | Up | 2.06 | 1.04 | 0.0482 |
|  |  | ko:K01321 | comp62328_c0_seq2 | | F9; coagulation factor IX (Christmas factor) [EC:3.4.21.22] | | | | | Up | 3.99 | 2.00 | 0.0003 |
|  |  | ko:K01321 | comp64057_c0_seq1 | | F9; coagulation factor IX (Christmas factor) [EC:3.4.21.22] | | | | | Up | 2.16 | 1.11 | 0.0379 |
|  |  | ko:K01330 | comp58616_c0_seq2 | | C1R; complement component 1, r subcomponent [EC:3.4.21.41] | | | | | Up | 2.79 | 1.48 | 0.0051 |
|  |  | ko:K01332 | comp67844_c1_seq9 | | C2; complement component 2 [EC:3.4.21.43] | | | | | Up | 2.45 | 1.29 | 0.0148 |
|  |  | ko:K01333 | comp64792_c0_seq2 | | CFI; complement factor I [EC:3.4.21.45] | | | | | Up | 5.33 | 2.41 | 0.0000 |
|  |  | ko:K01335 | comp14093_c0_seq1 | | CFB; component factor B [EC:3.4.21.47] | | | | | Up | 3.66 | 1.87 | 0.0005 |
|  |  | ko:K01344 | comp51226_c0_seq1 | | PROC; protein C (activated) [EC:3.4.21.69] | | | | | Up | 2.84 | 1.50 | 0.0051 |
|  |  | ko:K03898 | comp65879_c0_seq1 | | KNG; kininogen | | | | | Up | 2.44 | 1.29 | 0.0147 |
|  |  | ko:K03903 | comp63621_c1_seq6 | | FGA; fibrinogen alpha chain | | | | | Up | 2.20 | 1.14 | 0.0294 |
|  |  | ko:K03904 | comp54730_c0_seq1 | | FGB; fibrinogen beta chain | | | | | Up | 2.12 | 1.08 | 0.0383 |
|  |  | ko:K03905 | comp40601_c0_seq1 | | FGG; fibrinogen gamma chain | | | | | Up | 2.78 | 1.48 | 0.0052 |
|  |  | ko:K03908 | comp70226_c1_seq1 | | PROS1; protein S | | | | | Up | 2.07 | 1.05 | 0.0489 |
|  |  | ko:K03912 | comp63845_c0_seq1 | | SERPIND1, HCF2; heparin cofactor II | | | | | Up | 2.17 | 1.12 | 0.0339 |
|  |  | ko:K03983 | comp63530_c1_seq1 | | SERPINF2, AAP; alpha-2-antiplasmin | | | | | Up | 3.55 | 1.83 | 0.0007 |
|  |  | ko:K03990 | comp57282_c0_seq1 | | C3; complement component 3 | | | | | Up | 3.79 | 1.92 | 0.0003 |
|  |  | ko:K03990 | comp64668_c3_seq4 | | C3; complement component 3 | | | | | Up | 2.30 | 1.20 | 0.0245 |
|  |  | ko:K03992 | comp57583_c0_seq1 | | MASP1; mannan-binding lectin serine protease 1 [EC:3.4.21.-] | | | | | Up | 2.67 | 1.41 | 0.0219 |
|  |  | ko:K03992 | comp65138_c2_seq5 | | MASP1; mannan-binding lectin serine protease 1 [EC:3.4.21.-] | | | | | Up | 6.28 | 2.65 | 0.0013 |
|  |  | ko:K03994 | comp54789_c0_seq1 | | C5; complement component 5 | | | | | Up | 3.40 | 1.77 | 0.0009 |
|  |  | ko:K03996 | comp64828_c0_seq1 | | C7; complement component 7 | | | | | Down | 0.41 | -1.28 | 0.0286 |
|  |  | ko:K03996 | comp71185_c1_seq1 | | C7; complement component 7 | | | | | Up | 17.58 | 4.14 | 0.0000 |
|  |  | ko:K03997 | comp65864_c0_seq1 | | C8A; complement component 8 subunit alpha | | | | | Up | 4.15 | 2.05 | 0.0001 |
|  |  | ko:K03998 | comp62206_c0_seq1 | | C8B; complement component 8 subunit beta | | | | | Up | 4.83 | 2.27 | 0.0000 |
|  |  | ko:K03999 | comp60438_c0_seq1 | | C8G; complement component 8 subunit gamma | | | | | Up | 3.83 | 1.94 | 0.0003 |
|  |  | ko:K04000 | comp66790_c0_seq2 | | C9; complement component 9 | | | | | Up | 10.32 | 3.37 | 0.0000 |
|  |  | ko:K04001 | comp61753_c0_seq1 | | SERPING1, C1INH; C1 inhibitor | | | | | Up | 2.89 | 1.53 | 0.0038 |
|  |  | ko:K04004 | comp67044_c0_seq7 | | HF1; complement factor H | | | | | Up | 4.55 | 2.19 | 0.0001 |
|  |  | ko:K04008 | comp18297_c0_seq1 | | CD59; CD59 antigen | | | | | Up | 3.39 | 1.76 | 0.0242 |
|  |  | ko:K04008 | comp54684_c0_seq1 | | CD59; CD59 antigen | | | | | Up | 3.63 | 1.86 | 0.0006 |
|  |  | ko:K04008 | comp57062_c0_seq1 | | CD59; CD59 antigen | | | | | Up | 4.18 | 2.06 | 0.0002 |
| **ko04611** | **Platelet activation** | | | |  | | | | |  |  |  |  |
|  |  | ko:K03903 | comp63621_c1_seq6 | | FGA; fibrinogen alpha chain | | | | | Up | 2.20 | 1.14 | 0.0294 |
|  |  | ko:K03904 | comp54730_c0_seq1 | | FGB; fibrinogen beta chain | | | | | Up | 2.12 | 1.08 | 0.0383 |
|  |  | ko:K03905 | comp40601_c0_seq1 | | FGG; fibrinogen gamma chain | | | | | Up | 2.78 | 1.48 | 0.0052 |
|  |  | ko:K06261 | comp67565_c7_seq4 | | GP1BA; platelet glycoprotein Ib alpha chain | | | | | Up | 2.39 | 1.26 | 0.0184 |
|  |  | ko:K07532 | comp71523_c0_seq1 | | ARHGEF12, LARG; Rho guanine nucleotide exchange factor 12 | | | | | Down | 0.35 | -1.53 | 0.0142 |
|  |  | ko:K16342 | comp67569_c2_seq20 | | PLA2G4, CPLA2; cytosolic phospholipase A2 [EC:3.1.1.4] | | | | | Down | 0.20 | -2.33 | 0.0001 |
| **ko04620** | **Toll-like receptor signaling pathway** | | | |  | | | | |  |  |  |  |
|  |  | ko:K04379 | comp66390_c3_seq1 | | FOS; proto-oncogene protein c-fos | | | | | Up | 2.20 | 1.14 | 0.0359 |
|  |  | ko:K04734 | comp65648_c0_seq1 | | NFKBIA; NF-kappa-B inhibitor alpha | | | | | Down | 0.48 | -1.05 | 0.0482 |
|  |  | ko:K05398 | comp6353_c0_seq1 | | TLR1; toll-like receptor 1 | | | | | Down | 0.26 | -1.93 | 0.0347 |
|  |  | ko:K05398 | comp69977_c1_seq4 | | TLR1; toll-like receptor 1 | | | | | Down | 0.30 | -1.76 | 0.0028 |
|  |  | ko:K05425 | comp16585_c0_seq1 | | IL12B; interleukin 12B | | | | | Down | 0.05 | -4.26 | 0.0239 |
|  |  | ko:K10030 | comp51351_c0_seq5 | | IL8, CXCL8; interleukin 8 | | | | | Down | 0.29 | -1.78 | 0.0070 |
|  |  | ko:K10030 | comp63378_c0_seq16 | | IL8, CXCL8; interleukin 8 | | | | | Down | 0.13 | -2.96 | 0.0102 |
|  |  | ko:K10159 | comp64223_c0_seq1 | | TLR2; toll-like receptor 2 | | | | | Down | 0.35 | -1.50 | 0.0125 |
|  |  | ko:K12671 | comp50472_c0_seq1 | | CXCL10, IP10; C-X-C motif chemokine 10 | | | | | Down | 0.45 | -1.15 | 0.0454 |
|  |  | ko:K12671 | comp66168_c0_seq1 | | CXCL10, IP10; C-X-C motif chemokine 10 | | | | | Down | 0.38 | -1.39 | 0.0120 |
|  |  | ko:K12964 | comp60517_c1_seq2 | | CCL4; C-C motif chemokine 4 | | | | | Down | 0.34 | -1.57 | 0.0059 |
| **ko04621** | **NOD-like receptor signaling pathway** | | | | | | | | |  |  |  |  |
|  |  | ko:K04734 | comp65648_c0_seq1 | | NFKBIA; NF-kappa-B inhibitor alpha | | | | | Down | 0.48 | -1.05 | 0.0482 |
|  |  | ko:K10030 | comp51351_c0_seq5 | | IL8, CXCL8; interleukin 8 | | | | | Down | 0.29 | -1.78 | 0.0070 |
|  |  | ko:K10030 | comp63378_c0_seq16 | | IL8, CXCL8; interleukin 8 | | | | | Down | 0.13 | -2.96 | 0.0102 |
| **ko04622** | **RIG-I-like receptor signaling pathway** | | | |  | | | | |  |  |  |  |
|  |  | ko:K04734 | comp65648_c0_seq1 | | NFKBIA; NF-kappa-B inhibitor alpha | | | | | Down | 0.48 | -1.05 | 0.0482 |
|  |  | ko:K05425 | comp16585_c0_seq1 | | IL12B; interleukin 12B | | | | | Down | 0.05 | -4.26 | 0.0239 |
|  |  | ko:K10030 | comp51351_c0_seq5 | | IL8, CXCL8; interleukin 8 | | | | | Down | 0.29 | -1.78 | 0.0070 |
|  |  | ko:K10030 | comp63378_c0_seq16 | | IL8, CXCL8; interleukin 8 | | | | | Down | 0.13 | -2.96 | 0.0102 |
|  |  | ko:K12671 | comp50472_c0_seq1 | | CXCL10, IP10; C-X-C motif chemokine 10 | | | | | Down | 0.45 | -1.15 | 0.0454 |
|  |  | ko:K12671 | comp66168_c0_seq1 | | CXCL10, IP10; C-X-C motif chemokine 10 | | | | | Down | 0.38 | -1.39 | 0.0120 |
| **ko04623** | **Cytosolic DNA-sensing pathway** | | | | | | | | |  |  |  |  |
|  |  | ko:K04734 | comp65648_c0_seq1 | | NFKBIA; NF-kappa-B inhibitor alpha | | | | | Down | 0.48 | -1.05 | 0.0482 |
|  |  | ko:K12671 | comp50472_c0_seq1 | | CXCL10, IP10; C-X-C motif chemokine 10 | | | | | Down | 0.45 | -1.15 | 0.0454 |
|  |  | ko:K12671 | comp66168_c0_seq1 | | CXCL10, IP10; C-X-C motif chemokine 10 | | | | | Down | 0.38 | -1.39 | 0.0120 |
|  |  | ko:K12964 | comp60517_c1_seq2 | | CCL4; C-C motif chemokine 4 | | | | | Down | 0.34 | -1.57 | 0.0059 |
| **ko04650** | **Natural killer cell mediated cytotoxicity** | | | |  | | | | |  |  |  |  |
|  |  | ko:K06453 | comp17076_c0_seq1 | | CD3Z; CD3Z antigen, zeta polypeptide | | | | | Down | 0.36 | -1.48 | 0.0120 |
|  |  | ko:K17447 | comp69980_c0_seq5 | | SHC2; SHC- transforming protein 2 | | | | | Up | 2.20 | 1.13 | 0.0435 |
| **ko04612** | **Antigen processing and presentation** | | | | | | | | |  |  |  |  |
|  |  | ko:K06454 | comp70556_c0_seq2 | | CD4; CD4 antigen | | | | | Down | 0.41 | -1.29 | 0.0301 |
|  |  | ko:K06458 | comp68785_c0_seq8 | | CD8A; CD8A antigen, alpha polypeptide | | | | | Down | 0.38 | -1.38 | 0.0198 |
|  |  | ko:K08060 | comp64332_c0_seq1 | | CIITA; class II, major histocompatibility complex, transactivator | | | | | Down | 0.41 | -1.28 | 0.0297 |
| **ko04660** | **T cell receptor signaling pathway** | | | | | | | | |  |  |  |  |
|  |  | ko:K04379 | comp66390_c3_seq1 | | FOS; proto-oncogene protein c-fos | | | | | Up | 2.20 | 1.14 | 0.0359 |
|  |  | ko:K04734 | comp65648_c0_seq1 | | NFKBIA; NF-kappa-B inhibitor alpha | | | | | Down | 0.48 | -1.05 | 0.0482 |
|  |  | ko:K06453 | comp17076_c0_seq1 | | CD3Z; CD3Z antigen, zeta polypeptide | | | | | Down | 0.36 | -1.48 | 0.0120 |
|  |  | ko:K06454 | comp70556_c0_seq2 | | CD4; CD4 antigen | | | | | Down | 0.41 | -1.29 | 0.0301 |
|  |  | ko:K06458 | comp68785_c0_seq8 | | CD8A; CD8A antigen, alpha polypeptide | | | | | Down | 0.38 | -1.38 | 0.0198 |
| **ko04662** | **B cell receptor signaling pathway** | | | | | | | | |  |  |  |  |
|  |  | ko:K04379 | comp66390_c3_seq1 | | FOS; proto-oncogene protein c-fos | | | | | Up | 2.20 | 1.14 | 0.0359 |
|  |  | ko:K04734 | comp65648_c0_seq1 | | NFKBIA; NF-kappa-B inhibitor alpha | | | | | Down | 0.48 | -1.05 | 0.0482 |
|  |  | ko:K06467 | comp70355_c2_seq3 | | CD22, SIGLEC2; CD22 antigen | | | | | Down | 0.48 | -1.07 | 0.0497 |
|  |  | ko:K06507 | comp53870_c0_seq1 | | CD79B, IGB; CD79B antigen | | | | | Down | 0.41 | -1.28 | 0.0249 |
| **ko04664** | **Fc epsilon RI signaling pathway** | | | | | | | | |  |  |  |  |
|  |  | ko:K16342 | comp67569_c2_seq20 | | PLA2G4, CPLA2; cytosolic phospholipase A2 [EC:3.1.1.4] | | | | | Down | 0.20 | -2.33 | 0.0001 |
| **ko04666** | **Fc gamma R-mediated phagocytosis** | | | | | | | | |  |  |  |  |
|  |  | ko:K06498 | comp56824_c0_seq1 | | FCGR1A, CD64; high affinity immunoglobulin gamma Fc receptor I | | | | | Down | 0.48 | -1.06 | 0.0446 |
|  |  | ko:K12561 | comp63633_c0_seq1 | | MARCKS; myristoylated alanine-rich C-kinase substrate | | | | | Down | 0.46 | -1.12 | 0.0378 |
| **ko04670** | **Leukocyte transendothelial migration** | | | |  | | | | |  |  |  |  |
|  |  | ko:K06514 | comp30604_c0_seq1 | | THY1, CD90; Thy-1 cell surface antigen | | | | | Down | 0.12 | -3.02 | 0.0001 |
| **ko04672** | **Intestinal immune network for IgA production** | | | |  | | | | |  |  |  |  |
| **ko04062** | **Chemokine signaling pathway** | | | | | | | | |  |  |  |  |
|  |  | ko:K04193 | comp65787_c1_seq4 | | XCR1; XC chemokine receptor 1 | | | | | Down | 0.36 | -1.47 | 0.0106 |
|  |  | ko:K04734 | comp65648_c0_seq1 | | NFKBIA; NF-kappa-B inhibitor alpha | | | | | Down | 0.48 | -1.05 | 0.0482 |
|  |  | ko:K05512 | comp51567_c0_seq1 | | CCL19, ELC; C-C motif chemokine 19 | | | | | Down | 0.29 | -1.80 | 0.0008 |
|  |  | ko:K05512 | comp59357_c0_seq1 | | CCL19, ELC; C-C motif chemokine 19 | | | | | Down | 0.13 | -2.91 | 0.0029 |
|  |  | ko:K10030 | comp51351_c0_seq5 | | IL8, CXCL8; interleukin 8 | | | | | Down | 0.29 | -1.78 | 0.0070 |
|  |  | ko:K10030 | comp63378_c0_seq16 | | IL8, CXCL8; interleukin 8 | | | | | Down | 0.13 | -2.96 | 0.0102 |
|  |  | ko:K10032 | comp58804_c0_seq1 | | CXCL13; C-X-C motif chemokine 13 | | | | | Down | 0.29 | -1.77 | 0.0079 |
|  |  | ko:K12671 | comp50472_c0_seq1 | | CXCL10, IP10; C-X-C motif chemokine 10 | | | | | Down | 0.45 | -1.15 | 0.0454 |
|  |  | ko:K12671 | comp66168_c0_seq1 | | CXCL10, IP10; C-X-C motif chemokine 10 | | | | | Down | 0.38 | -1.39 | 0.0120 |
|  |  | ko:K12964 | comp60517_c1_seq2 | | CCL4; C-C motif chemokine 4 | | | | | Down | 0.34 | -1.57 | 0.0059 |
|  |  | ko:K17447 | comp69980_c0_seq5 | | SHC2; SHC- transforming protein 2 | | | | | Up | 2.20 | 1.13 | 0.0435 |
|  |  |  |  | |  | | | | |  |  |  |  |
| **E vs A** |  |  |  | |  | | | | |  |  |  |  |
| **ko04640** | **Hematopoietic cell lineage** | | | | | | | | |  |  |  |  |
|  |  | ko:K04008 | comp54684_c0_seq1 | | CD59; CD59 antigen | | | | | Up | 5.31 | 2.41 | 0.0000 |
|  |  | ko:K04008 | comp57062_c0_seq1 | | CD59; CD59 antigen | | | | | Up | 7.43 | 2.89 | 0.0000 |
|  |  | ko:K04519 | comp24971_c0_seq1 | | IL1B; interleukin 1 beta | | | | | Up | 3.26 | 1.70 | 0.0111 |
|  |  | ko:K05423 | comp60827_c2_seq1 | | CSF3, GCSF; granulocyte colony-stimulating factor | | | | | Up | 6.63 | 2.73 | 0.0012 |
|  |  | ko:K06449 | comp68860_c0_seq1 | | CD2; CD2 antigen | | | | | Down | 0.26 | -1.93 | 0.0038 |
|  |  | ko:K06467 | comp70355_c2_seq3 | | CD22, SIGLEC2; CD22 antigen | | | | | Down | 0.33 | -1.58 | 0.0044 |
|  |  | ko:K06481 | comp70612_c1_seq1 | | ITGA2; integrin alpha 2 | | | | | Down | 0.41 | -1.30 | 0.0496 |
|  |  | ko:K06503 | comp64406_c3_seq4 | | TFRC, CD71; transferrin receptor | | | | | Up | 2.99 | 1.58 | 0.0046 |
| **ko04610** | **Complement and coagulation cascades** | | | | | | | | |  |  |  |  |
|  |  | ko:K01300 | comp65823_c0_seq1 | | CPB2; carboxypeptidase B2 [EC:3.4.17.20] | | | | | Up | 4.40 | 2.14 | 0.0001 |
|  |  | ko:K01314 | comp45692_c0_seq2 | | F10; coagulation factor X [EC:3.4.21.6] | | | | | Up | 2.43 | 1.28 | 0.0157 |
|  |  | ko:K01315 | comp58611_c0_seq1 | | PLG; plasminogen [EC:3.4.21.7] | | | | | Up | 3.97 | 1.99 | 0.0002 |
|  |  | ko:K01320 | comp43168_c0_seq1 | | F7; coagulation factor VII [EC:3.4.21.21] | | | | | Up | 2.31 | 1.21 | 0.0244 |
|  |  | ko:K01320 | comp54518_c0_seq1 | | F7; coagulation factor VII [EC:3.4.21.21] | | | | | Up | 3.54 | 1.82 | 0.0009 |
|  |  | ko:K01320 | comp57677_c0_seq1 | | F7; coagulation factor VII [EC:3.4.21.21] | | | | | Up | 2.11 | 1.08 | 0.0419 |
|  |  | ko:K01321 | comp62328_c0_seq2 | | F9; coagulation factor IX (Christmas factor) [EC:3.4.21.22] | | | | | Up | 3.32 | 1.73 | 0.0015 |
|  |  | ko:K01321 | comp64057_c0_seq1 | | F9; coagulation factor IX (Christmas factor) [EC:3.4.21.22] | | | | | Up | 3.36 | 1.75 | 0.0013 |
|  |  | ko:K01324 | comp66827_c0_seq1 | | KLKB1; plasma kallikrein [EC:3.4.21.34] | | | | | Up | 2.71 | 1.44 | 0.0069 |
|  |  | ko:K01330 | comp58616_c0_seq2 | | C1R; complement component 1, r subcomponent [EC:3.4.21.41] | | | | | Up | 2.29 | 1.20 | 0.0228 |
|  |  | ko:K01332 | comp67844_c1_seq9 | | C2; complement component 2 [EC:3.4.21.43] | | | | | Up | 2.34 | 1.23 | 0.0204 |
|  |  | ko:K01333 | comp64792_c0_seq2 | | CFI; complement factor I [EC:3.4.21.45] | | | | | Up | 5.26 | 2.39 | 0.0000 |
|  |  | ko:K01335 | comp14093_c0_seq1 | | CFB; component factor B [EC:3.4.21.47] | | | | | Up | 4.72 | 2.24 | 0.0000 |
|  |  | ko:K01344 | comp51226_c0_seq1 | | PROC; protein C (activated) [EC:3.4.21.69] | | | | | Up | 3.40 | 1.77 | 0.0011 |
|  |  | ko:K03898 | comp65879_c0_seq1 | | KNG; kininogen | | | | | Up | 4.48 | 2.16 | 0.0001 |
|  |  | ko:K03903 | comp63621_c1_seq6 | | FGA; fibrinogen alpha chain | | | | | Up | 2.08 | 1.06 | 0.0435 |
|  |  | ko:K03904 | comp54730_c0_seq1 | | FGB; fibrinogen beta chain | | | | | Up | 2.63 | 1.39 | 0.0083 |
|  |  | ko:K03905 | comp40601_c0_seq1 | | FGG; fibrinogen gamma chain | | | | | Up | 2.95 | 1.56 | 0.0033 |
|  |  | ko:K03908 | comp70226_c1_seq1 | | PROS1; protein S | | | | | Up | 2.27 | 1.18 | 0.0275 |
|  |  | ko:K03982 | comp65845_c0_seq1 | | SERPINE1, PAI1; plasminogen activator inhibitor-1 | | | | | Up | 3.21 | 1.68 | 0.0020 |
|  |  | ko:K03983 | comp51417_c0_seq1 | | SERPINF2, AAP; alpha-2-antiplasmin | | | | | Up | 2.68 | 1.42 | 0.0090 |
|  |  | ko:K03983 | comp63530_c1_seq1 | | SERPINF2, AAP; alpha-2-antiplasmin | | | | | Up | 3.15 | 1.65 | 0.0020 |
|  |  | ko:K03990 | comp57282_c0_seq1 | | C3; complement component 3 | | | | | Up | 3.34 | 1.74 | 0.0011 |
|  |  | ko:K03990 | comp64668_c3_seq4 | | C3; complement component 3 | | | | | Up | 2.16 | 1.11 | 0.0379 |
|  |  | ko:K03992 | comp65138_c2_seq5 | | MASP1; mannan-binding lectin serine protease 1 [EC:3.4.21.-] | | | | | Up | 3.40 | 1.77 | 0.0309 |
|  |  | ko:K03994 | comp54789_c0_seq1 | | C5; complement component 5 | | | | | Up | 2.59 | 1.37 | 0.0094 |
|  |  | ko:K03996 | comp71185_c1_seq1 | | C7; complement component 7 | | | | | Up | 8.05 | 3.01 | 0.0000 |
|  |  | ko:K03997 | comp65864_c0_seq1 | | C8A; complement component 8 subunit alpha | | | | | Up | 3.40 | 1.76 | 0.0010 |
|  |  | ko:K03998 | comp62206_c0_seq1 | | C8B; complement component 8 subunit beta | | | | | Up | 4.39 | 2.13 | 0.0001 |
|  |  | ko:K03999 | comp60438_c0_seq1 | | C8G; complement component 8 subunit gamma | | | | | Up | 3.26 | 1.70 | 0.0015 |
|  |  | ko:K04000 | comp66790_c0_seq2 | | C9; complement component 9 | | | | | Up | 6.83 | 2.77 | 0.0000 |
|  |  | ko:K04001 | comp61753_c0_seq1 | | SERPING1, C1INH; C1 inhibitor | | | | | Up | 2.69 | 1.43 | 0.0069 |
|  |  | ko:K04004 | comp67044_c0_seq7 | | HF1; complement factor H | | | | | Up | 4.79 | 2.26 | 0.0000 |
|  |  | ko:K04004 | comp68996_c0_seq3 | | HF1; complement factor H | | | | | Up | 3.15 | 1.66 | 0.0019 |
|  |  | ko:K04008 | comp54684_c0_seq1 | | CD59; CD59 antigen | | | | | Up | 5.31 | 2.41 | 0.0000 |
|  |  | ko:K04008 | comp57062_c0_seq1 | | CD59; CD59 antigen | | | | | Up | 7.43 | 2.89 | 0.0000 |
| **ko04611** | **Platelet activation** | | | |  | | | | |  |  |  |  |
|  |  | ko:K03903 | comp63621_c1_seq6 | | FGA; fibrinogen alpha chain | | | | | Up | 2.08 | 1.06 | 0.0435 |
|  |  | ko:K03904 | comp54730_c0_seq1 | | FGB; fibrinogen beta chain | | | | | Up | 2.63 | 1.39 | 0.0083 |
|  |  | ko:K03905 | comp40601_c0_seq1 | | FGG; fibrinogen gamma chain | | | | | Up | 2.95 | 1.56 | 0.0033 |
|  |  | ko:K04958 | comp70776_c0_seq5 | | ITPR1; inositol 1,4,5-triphosphate receptor type 1 | | | | | Down | 0.29 | -1.77 | 0.0049 |
|  |  | ko:K05719 | comp62046_c0_seq1 | | ITGB1; integrin beta 1 | | | | | Up | 2.37 | 1.24 | 0.0234 |
|  |  | ko:K06481 | comp70612_c1_seq1 | | ITGA2; integrin alpha 2 | | | | | Down | 0.41 | -1.30 | 0.0496 |
|  |  | ko:K08041 | comp25107_c0_seq1 | | ADCY1; adenylate cyclase 1 [EC:4.6.1.1] | | | | | Up | 10.33 | 3.37 | 0.0164 |
|  |  | ko:K08042 | comp65282_c1_seq2 | | ADCY2; adenylate cyclase 2 [EC:4.6.1.1] | | | | | Down | 0.31 | -1.71 | 0.0170 |
|  |  | ko:K16342 | comp67569_c2_seq20 | | PLA2G4, CPLA2; cytosolic phospholipase A2 [EC:3.1.1.4] | | | | | Down | 0.26 | -1.94 | 0.0008 |
| **ko04620** | **Toll-like receptor signaling pathway** | | | |  | | | | |  |  |  |  |
|  |  | ko:K04519 | comp24971_c0_seq1 | | IL1B; interleukin 1 beta | | | | | Up | 3.26 | 1.70 | 0.0111 |
|  |  | ko:K05398 | comp69977_c1_seq4 | | TLR1; toll-like receptor 1 | | | | | Down | 0.35 | -1.53 | 0.0088 |
|  |  | ko:K10168 | comp68815_c0_seq1 | | TLR5; toll-like receptor 5 | | | | | Up | 5.08 | 2.34 | 0.0000 |
| **ko04621** | **NOD-like receptor signaling pathway** | | | | | | | | |  |  |  |  |
|  |  | ko:K04519 | comp24971_c0_seq1 | | IL1B; interleukin 1 beta | | | | | Up | 3.26 | 1.70 | 0.0111 |
|  |  | ko:K09487 | comp58660_c1_seq1 | | HSP90B, TRA1; heat shock protein 90kDa beta | | | | | Up | 2.10 | 1.07 | 0.0407 |
| **ko04622** | **RIG-I-like receptor signaling pathway** | | | |  | | | | |  |  |  |  |
| **ko04623** | **Cytosolic DNA-sensing pathway** | | | | | | | | |  |  |  |  |
|  |  | ko:K04519 | comp24971_c0_seq1 | | IL1B; interleukin 1 beta | | | | | Up | 3.26 | 1.70 | 0.0111 |
| **ko04650** | **Natural killer cell mediated cytotoxicity** | | | |  | | | | |  |  |  |  |
|  |  | ko:K01353 | comp69662_c0_seq5 | | GZMB; granzyme B [EC:3.4.21.79] | | | | | Down | 0.39 | -1.36 | 0.0309 |
|  |  | ko:K17447 | comp69980_c0_seq5 | | SHC2; SHC- transforming protein 2 | | | | | Up | 2.53 | 1.34 | 0.0178 |
| **ko04612** | **Antigen processing and presentation** | | | | | | | | |  |  |  |  |
|  |  | ko:K01365 | comp51540_c0_seq1 | | CTSL; cathepsin L [EC:3.4.22.15] | | | | | Up | 2.37 | 1.24 | 0.0188 |
|  |  | ko:K03283 | comp57103_c2_seq3 | | HSPA1_8; heat shock 70kDa protein 1/8 | | | | | Up | 2.19 | 1.13 | 0.0321 |
| **ko04660** | **T cell receptor signaling pathway** | | | | | | | | |  |  |  |  |
| **ko04662** | **B cell receptor signaling pathway** | | | | | | | | |  |  |  |  |
|  |  | ko:K06467 | comp70355_c2_seq3 | | CD22, SIGLEC2; CD22 antigen | | | | | Down | 0.33 | -1.58 | 0.0044 |
|  |  | ko:K06507 | comp53870_c0_seq1 | | CD79B, IGB; CD79B antigen | | | | | Down | 0.34 | -1.56 | 0.0068 |
| **ko04664** | **Fc epsilon RI signaling pathway** | | | | | | | | |  |  |  |  |
|  |  | ko:K16342 | comp67569_c2_seq20 | | PLA2G4, CPLA2; cytosolic phospholipase A2 [EC:3.1.1.4] | | | | | Down | 0.26 | -1.94 | 0.0008 |
| **ko04666** | **Fc gamma R-mediated phagocytosis** | | | | | | | | |  |  |  |  |
|  |  | ko:K05768 | comp61214_c0_seq12 | | GSN; gelsolin | | | | | Down | 0.38 | -1.41 | 0.0250 |
| **ko04670** | **Leukocyte transendothelial migration** | | | |  | | | | |  |  |  |  |
|  |  | ko:K04189 | comp64098_c0_seq1 | | CXCR4; C-X-C chemokine receptor type 4 | | | | | Up | 3.08 | 1.62 | 0.0283 |
|  |  | ko:K05719 | comp62046_c0_seq1 | | ITGB1; integrin beta 1 | | | | | Up | 2.37 | 1.24 | 0.0234 |
|  |  | ko:K06087 | comp56654_c0_seq1 | | CLDN; claudin | | | | | Up | 2.84 | 1.51 | 0.0061 |
|  |  | ko:K06514 | comp30604_c0_seq1 | | THY1, CD90; Thy-1 cell surface antigen | | | | | Down | 0.25 | -2.02 | 0.0052 |
| **ko04672** | **Intestinal immune network for IgA production** | | | |  | | | | |  |  |  |  |
|  |  | ko:K04189 | comp64098_c0_seq1 | | CXCR4; C-X-C chemokine receptor type 4 | | | | | Up | 3.08 | 1.62 | 0.0283 |
| **ko04062** | **Chemokine signaling pathway** | | | | | | | | |  |  |  |  |
|  |  | ko:K04189 | comp64098_c0_seq1 | | CXCR4; C-X-C chemokine receptor type 4 | | | | | Up | 3.08 | 1.62 | 0.0283 |
|  |  | ko:K04190 | comp66135_c0_seq7 | | CXCR5, BLR1; C-X-C chemokine receptor type 5 | | | | | Down | 0.39 | -1.36 | 0.0247 |
|  |  | ko:K04347 | comp65261_c6_seq1 | | GNG12; guanine nucleotide-binding protein G(I)/G(S)/G(O) subunit gamma-12 | | | | | Up | 2.56 | 1.35 | 0.0109 |
|  |  | ko:K05050 | comp71229_c1_seq1 | | IL8RB, CXCR2; interleukin 8 receptor beta | | | | | Down | 0.17 | -2.54 | 0.0040 |
|  |  | ko:K08041 | comp25107_c0_seq1 | | ADCY1; adenylate cyclase 1 [EC:4.6.1.1] | | | | | Up | 10.33 | 3.37 | 0.0164 |
|  |  | ko:K08042 | comp65282_c1_seq2 | | ADCY2; adenylate cyclase 2 [EC:4.6.1.1] | | | | | Down | 0.31 | -1.71 | 0.0170 |
|  |  | ko:K17447 | comp69980_c0_seq5 | | SHC2; SHC- transforming protein 2 | | | | | Up | 2.53 | 1.34 | 0.0178 |
